# Long‐Term Safety of Desmopressin Orally Disintegrating Tablets in Men With Nocturia due to Nocturnal Polyuria: Final Results of a Specified Drug Use‐Results Survey in Japan

**DOI:** 10.1111/luts.70052

**Published:** 2026-03-17

**Authors:** Yoshimasa Ogawa, Kiyotoshi Kuramoto, Atsushi Nakano

**Affiliations:** ^1^ Post‐Marketing Surveillance, Medical Affairs Ferring Pharmaceuticals Co. Ltd. Tokyo Japan; ^2^ Pharmacovigilance & Post‐Marketing Surveillance Kissei Pharmaceutical Co. Ltd. Tokyo Japan; ^3^ Medical Affairs Ferring Pharmaceuticals Co. Ltd. Tokyo Japan

## Abstract

**Objectives:**

This report presents the final results of a post‐marketing surveillance evaluating the safety of desmopressin orally disintegrating tablets (ODT) in Japanese men with nocturia due to nocturnal polyuria (NP).

**Methods:**

In total, 1113 Japanese men who received desmopressin ODT for the first time to treat nocturia due to NP were enrolled in a central registry. Of the 1087 patients for which case report forms were collected, 38 did not meet the safety eligibility criteria of the survey. Consequently, the remaining 1049 patients were included in the analysis. The participants were followed for up to 52 weeks.

**Results:**

The mean age of the total population was 75.0 ± 9.6 years. A total of 351 adverse drug reactions (ADRs) were reported in 259 patients, including 11 serious ADRs in 7 patients (0.7%). The most common ADR was hyponatremia, which occurred in 140 patients (13.3%). Among the 19 patients (13.6%) presenting hyponatremia related symptoms, no reports of confusion, seizures, stupor, or coma was recorded. Multivariate analysis identified age ≥ 75 years, a history of benign prostatic hyperplasia, and a lower serum sodium level before treatment as risk factors for the development of hypernatremia after the treatment with desmopressin ODT.

**Conclusions:**

The survey provides insights into the safety profile of desmopressin ODT in real‐clinical practice in Japan. Patients aged ≥ 75 years, those with underlying medical conditions, and those with abnormal clinical laboratory values, such as reduced renal function or a baseline serum sodium level of < 140 mmol/L, should be closely monitored for the development of hyponatremia during desmopressin treatment for nocturia due to NP.

## 1 Introduction

Nocturia becomes more prevalent with advancing age, reducing sleep quality and overall quality of life, and increasing the risk of nighttime falls and fractures, leading to concern about its negative prognostic impact [1]. Nocturia is associated with reduced secretion of arginine vasopressin, also known as antidiuretic hormone [2], and nocturnal polyuria (NP) is a major contributor [3].

Desmopressin is a synthetic peptide derivative of arginine vasopressin that exerts minimal effects on blood pressure but suppresses urine production, and demonstrates a prolonged dose‐dependent antidiuretic effect [4, 5]. However, desmopressin is associated with a risk of hyponatremia due to its effect of excessive water retention induced by its antidiuretic effect [6]. In general, serum sodium concentrations of 125 mEq/L or higher are asymptomatic (though sometimes accompanied by headache, nausea, or memory decline), 120–125 mEq/L may cause confusion or loss of appetite, 115–120 mEq/L may lead to restlessness, somnolence, or stupor, and concentrations below 115 mEq/L may result in convulsions or coma. Reports indicate a mortality rate of 30% at 115–120 mEq/L and 50% below 115 mEq/L [7]. Therefore, early recognition of the initial symptoms of hyponatremia (e.g., malaise, headache, nausea, and vomiting) is essential during the treatment with desmopressin. Desmopressin‐induced hyponatremia is typically mild and reversible, but it may compromise the treatment of elderly patients with nocturia [8, 9, 10].

Desmopressin orally disintegrating tablets (ODT, 25 and 50 μg; MINIRINMELT; Ferring Pharmaceuticals, Parsippany, NJ) were approved in Japan in June 2019 and launched in September 2019 for the treatment of nocturia due to NP in men. Fifty micrograms of desmopressin ODT is administered orally once daily before going to bed in male adults, but starting with 25 μg of desmopressin ODT should be considered based on the patient's condition such as age, weight, level of serum sodium, or cardiac function due to the risk of developing hyponatremia. In Japan, manufacturers and marketing authorization holders are required to conduct post‐marketing surveillance (PMS) for a specified period after drug approval allowing the Ministry of Health, Labour and Welfare to continuously evaluate its efficacy and safety [11]. Ferring Japan has previously reported interim PMS results on the safety of long‐term use of desmopressin ODT in 300 patients included in the safety analysis set, followed for up to 12 weeks post‐treatment [11]. The present report provides the final safety findings in 1049 patients followed for up to 52 weeks.

## 2 Methods

This PMS was conducted as part of the desmopressin surveillance program and in accordance with the Good Post‐marketing Study Practice standards established by the Japanese Ministry of Health, Labour and Welfare [11]. The survey is registered on ClinicalTrials.gov (NCT04329975). In this survey, hyponatremia was defined based on the clinical judgment of attending physicians. Physicians were not required to submit laboratory data on serum sodium levels or to make diagnoses based on the data.

### 2.1 Study Design

This survey was a prospective, observational, multicenter PMS involving Japanese men with physician‐diagnosed nocturia due to NP who had not previously been treated with desmopressin ODT. Patients were registered, and data were collected using an electronic data capture system. Each patient was enrolled by a physician within 1 week of starting desmopressin ODT (excluding the day of initiation). At the end of the observation period or at the time of treatment discontinuation, the physicians entered the survey data for all registered patients, including those who discontinued or withdrew from treatment. The survey was conducted via a central registry. The registration period was from March 2020 to the end of March 2022, at which point the target of enrolling 1000 patients for safety analysis set was met. The overall survey period, including the follow‐up period, lasted from March 2020 to the end of September 2023. The follow‐up duration was up to 52 weeks after treatment initiation. If treatment was discontinued, including completion of treatment as a result of symptom improvement, the observation period ended on the date of discontinuation. If a patient could not be followed until the end of the observation period due to transfer to another hospital or death, the observation period was considered to have ended at the point when further observation was no longer possible.

### 2.2 Study Assessments

Information was collected on patient characteristics, including background data, records of desmopressin ODT administration, status at the end of the observation period, concomitant medications and therapy for nocturia, laboratory test results, follow‐up for nocturia, adverse events (AEs), hyponatremia and its cause and symptoms if hyponatremia developed. Patient characteristics included diagnosis, height and body weight, creatinine clearance (Ccr) estimated by the Cockcroft–Gault equation, hemoglobin level, history of smoking and alcohol consumption, performance status, medical history, and comorbidities such as renal impairment, benign prostatic hyperplasia (BPH), overactive bladder, and interstitial cystitis.

The primary safety outcome measures include the incidence of desmopressin‐induced hyponatremia, distribution of serum sodium levels, time to onset of hyponatremia, and factors associated with its development. Hyponatremia was diagnosed by an investigator and categorized by severity based on serum sodium levels: mild (130–134 mmol/L), moderate (126–129 mmol/L), and severe (≤ 125 mmol/L).

An ADR was defined as an AE that, in the opinion of the investigator, was probably or possibly causally related to treatment with desmopressin ODT. All ADRs were summarized and coded using the Japanese Medical Dictionary for Regulatory Activities (MedDRA; version 26.0). An SAE was defined as any event that resulted in death, was life‐threatening, required inpatient hospitalization or prolongation of an existing hospitalization, resulted in persistent or significant disability/incapacity or congenital anomaly/birth defect, or was serious enough to have compromised patient safety or required medical or surgical intervention to prevent any of the above. The incidence of ADRs was evaluated for each background factor, including desmopressin ODT dose, patient age, baseline serum sodium level, renal function indicated by the Ccr, medical history, and concomitant medications.

### 2.3 Statistical Analysis

Actual clinical data were collected and tabulated without intervention. Missing values were treated as unknown or undescribed. Comparisons were performed using Fisher's exact test for two factors and the chi‐squared test for three or more factors. Ordinal scale data are expressed as the number and percentage. For continuous data, summary statistics were calculated and are expressed mainly as the mean ± standard deviation. The frequency of ADRs is expressed as a percentage, with the 1049 patients in the safety analysis set as the denominator and the number of ADRs as the numerator. An analysis stratified by patient background characteristics was performed to investigate potential risk factors for hyponatremia. To identify the factors that increased the risk of hyponatremia among those with significant results in the stratified analysis, we calculated the odds ratio (OR) and 95% confidence interval (CI) for each variable using multivariate logistic regression analysis. The Wald test was used for the independent variable elimination method. All statistical analyses were performed using SAS version 9.4 (SAS Institute, Cary, NC). All tests were two‐tailed, and a *p*‐value of < 0.05 was considered statistically significant.

## 3 Results

### 3.1 Study Population

**FIGURE 1:**
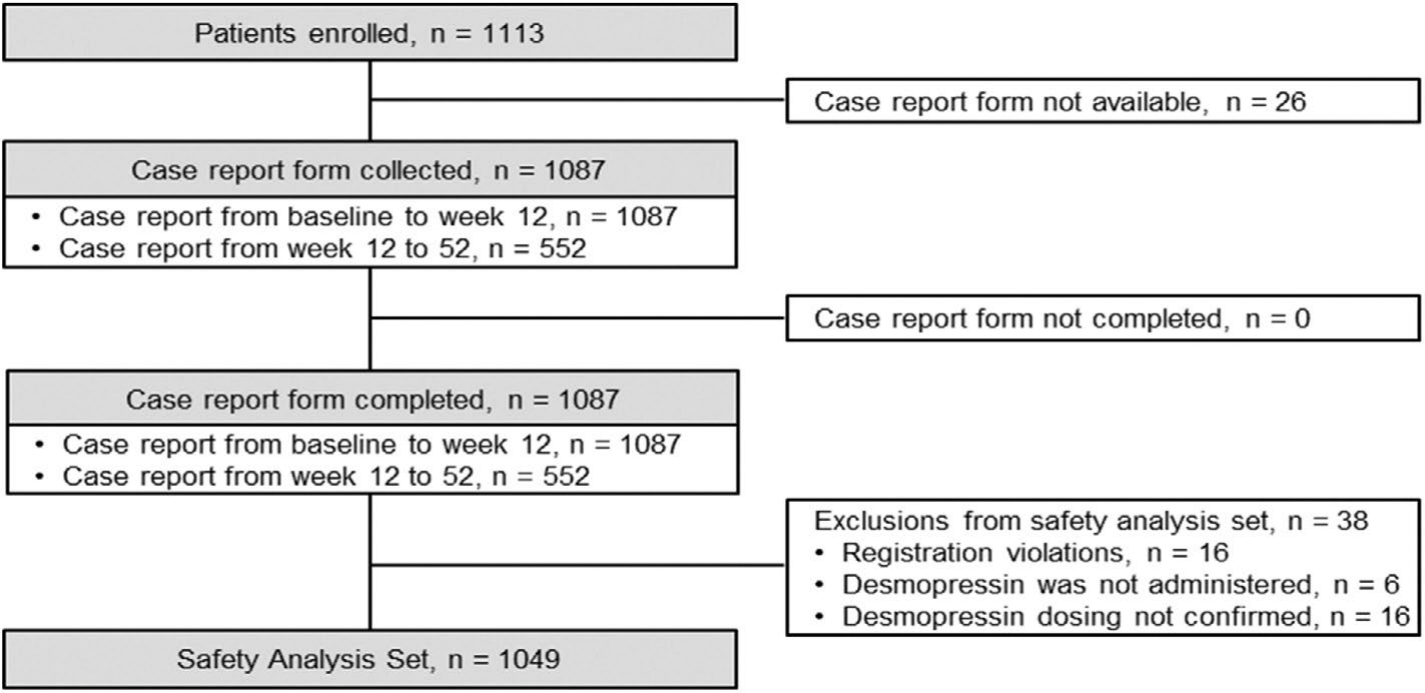
Flowchart of patients included in the survey. The safety analysis set comprised 1049 patients who had received desmopressin and for whom case report forms were collected by the end of March 2022.

In this survey, 1113 patients from 210 sites were registered, exceeding the planned target of 1000 patients. Twenty‐six patients were excluded due to uncollected case report forms. Of the 1087 patients for whom a case report form was collected (File S1, File S2), 38 were excluded for the following reasons: registration violation (*n* = 16), desmopressin not administered (*n* = 6), and desmopressin dosing not confirmed (*n* = 16). The remaining 1049 patients comprised the safety analysis set (Figure 1).

### 3.2 Baseline Patient Characteristics

Table 1 summarizes the baseline demographics and clinical characteristics of the patients. The mean age in the safety analysis set was 75.0 ± 9.6 years, with 60.0% of patients aged ≥ 75 years. The initial dose of desmopressin was 25 μg in 47.0% of total patients (493/1049). One patient was treated with desmopressin 50 μg at off‐label. Of the 424 patients in which the Cockcroft–Gault formula was used to calculate the estimated Ccr, 79 received desmopressin ODT despite having a pre‐treatment Ccr of < 50 mL/min. A comparison of patient background at the initial dose of 25 and 50 μg showed differences in age, body weight, Ccr estimate by Cockcroft–Gault equation before desmopressin, benign prostatic hyperplasia, overactive bladder, on medications for overactive bladder and concomitant medications for nocturia (including BPH and OAB).

**TABLE 1:** Baseline patient demographic and clinical characteristics/patient background of initial dose of desmopressin.

Characteristics		Total patients, *n* (%)	Initial dose of desmopressin		*χ* ^2^/Fisher‐test or *T*‐test^b^
			25 μg	50 μg	
			Patients, *n* (%)	Patients, *n* (%)	
Age (years) Range, 18–98 Mean ± SD, 75.0 ± 9.6	< 75	420 (40.0)	164 (33.3)	256 (46.0)	*p* < 0.000
	≥ 75	629 (60.0)	329 (66.7)	300 (54.0)	
Body weight (kg) Range, 30.0–107.6 Mean ± SD, 64.10 ± 9.96	< 40	3 (0.3)	3 (0.6)	0 (0.0)	*p* < 0.000
	40 to < 50	19 (1.8)	11 (2.2)	8 (1.4)	
	50 to < 60	147 (14.0)	69 (14.0)	78 (14.0)	
	60 to < 70	209 (19.9)	97 (19.7)	112 (20.1)	
	70 to < 80	109 (10.4)	40 (8.1)	69 (12.4)	
	≥ 80	31 (3.0)	6 (1.2)	25 (4.5)	
	Unknown	531 (50.6)	267 (54.2)	264 (47.5)	
Labeling	On‐label	1048 (99.9)	493 (100.0)	555 (99.8)	*p* = 0.346
	Off‐label	1 (0.1)	0 (0.0)	1 (0.2)	
Classification of consultations	Out	1039 (99.0)	486 (98.6)	553 (99.5)	*p* = 0.218
	Out‐in	3 (0.3)	3 (0.6)	0 (0.0)	
	Out‐in‐out	1 (0.1)	1 (0.2)	0 (0.0)	
	In	1 (0.1)	0 (0.0)	1 (0.2)	
	In‐out	5 (0.5)	3 (0.6)	2 (0.4)	
Initial dose of desmopressin (μg)	25	493 (47.0)			
	50	556 (53.0)			
Ccr estimated by Cockcroft–Gault equation before desmopressin (mL/min) Range, 27.8–157.7 Mean ± SD, 68.83 ± 22.16	< 30	2 (0.2)	2 (0.4)	0 (0.0)	*p* < 0.000
	30 to < 50	77 (7.3)	44 (8.9)	33 (5.9)	
	50 to < 80	242 (23.1)	109 (22.1)	133 (23.9)	
	≥ 80	103 (9.8)	27 (5.5)	76 (13.7)	
	NM	625 (59.6)	311 (63.1)	314 (56.5)	
Serum sodium level before desmopressin (mmol/L) Range, 125.0–155.0 Mean ± SD, 140.57 ± 2.57	< 126	1 (0.1)	0 (0.0)	1 (0.2)	*p* = 0.321
	126 to < 130	1 (0.1)	0 (0.0)	1 (0.2)	
	130 to < 135	8 (0.7)	6 (1.2)	2 (0.4)	
	135 to < 140	256 (24.4)	121 (24.5)	135 (24.3)	
	140 to < 148	602 (57.4)	274 (55.6)	328 (59.0)	
	≥ 148	3 (0.3)	0 (0.0)	3 (0.5)	
	NM	178 (17.0)	92 (18.7)	86 (15.5)	
BPH	No	341 (32.5)	139 (28.2)	202 (36.3)	*p* = 0.005
	Yes	708 (67.5)	354 (71.8)	354 (63.7)	
OAB	No	523 (49.9)	212 (43.0)	311 (55.9)	*p* < 0.000
	Yes	526 (50.1)	281 (57.0)	245 (44.1)	
On medication for BPH^a^	No	554 (52.8)	246 (49.9)	308 (55.4)	*p* = 0.075
	Yes	495 (47.2)	247 (50.1)	248 (44.6)	
On medication for OAB^a^	No	701 (66.8)	304 (61.7)	397 (71.4)	*p* = 0.001
	Yes	348 (33.2)	189 (38.3)	159 (28.6)	
On medication for nocturia (including BPH and OAB)	No	401 (38.2)	164 (33.3)	237 (42.6)	*p* = 0.002
	Yes	648 (61.8)	329 (66.7)	319 (57.4)	

Abbreviations: BPH, benign prostatic hyperplasia; Ccr, creatinine clearance; In, inpatient; NM, not measured; OAB, overactive bladder; Out, outpatient; SD, standard deviation.

^a^ BPH and OAB are determined by each physician's diagnosis.

^b^ *χ* ^2^/Fisher‐test between 25 and 50 μg: Labeling, classification of consultations, BPH, OAB, on medication for BPH, on medication for OAB, on medication for nocturia (including BPH and OAB). *T*‐test between 25 and 50 μg: Age, body weight, Ccr estimated by Cockcroft–Gault equation before desmopressin, serum sodium level before desmopressin.

### 3.3 Treatment Continuity

**FIGURE 2:**
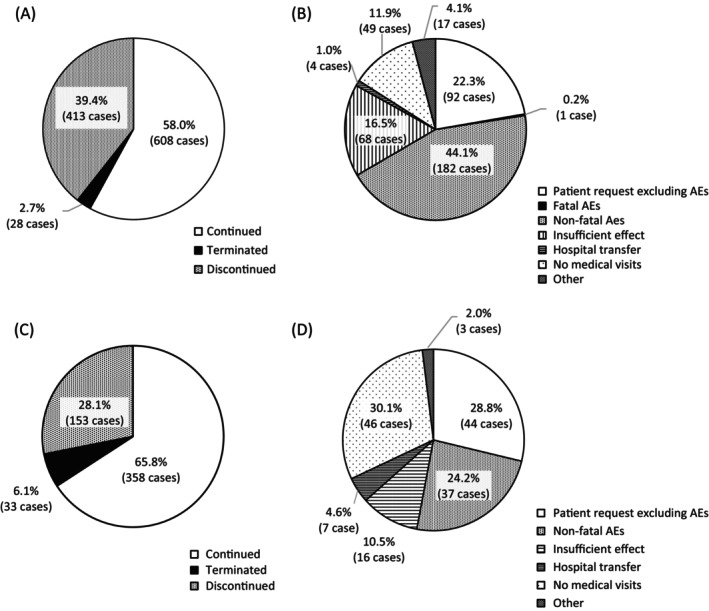
Desmopressin continuation status and reasons for discontinuation during the first 12 weeks of treatment (A, B) and during weeks 12–52 weeks (C, D).

During the first 12 weeks of treatment, desmopressin ODT was discontinued in 413 patients (39.4%) and terminated in 28 patients (2.7%) (Figure 2A). Reasons for discontinuation within the first 12 weeks included patient request without AEs in 92 patients (22.3%), fatal AEs in 1 (0.2%), non‐fatal AEs in 182 (44.1%), insufficient effect in 68 (16.5%), hospital transfer in 4 (1.0%), loss to follow‐up visits in 49 (11.9%), and other reasons in 17 (4.1%) (Figure 2B). Among the 544 patients whose CRF2 was collected and finalized, desmopressin ODT was discontinued in 153 patients (28.1%) and terminated in 33 (6.1%) between 12 and 52 weeks after treatment initiation (Figure 2C). Reasons for discontinuation during this period included patient request without AEs in 44 patients (28.8%), non‐fatal AEs in 37 (24.2%), insufficient effect in 16 (10.5%), hospital transfer in 7 (4.6%), loss to follow‐up visits in 46 (30.1%), and other reasons in 3 (2.0%), and no deaths due to AEs were observed (Figure 2D).

### 3.4 Safety

A total of 351 ADRs were reported in 259 patients, representing 24.7% of the safety analysis set (Table 2). Seven patients reported a total of 11 serious ADRs, including 4 patients with hyponatremia, 1 patient each with hypochloremia, cardiac failure congestive, hypertension, pleural effusion, cholecystitis acute, edema peripheral, and decreased glomerular filtration rate. One patient shows both cardiac failure congestive and decreased glomerular filtration rate. Another patient shows hyponatremia and pleural effusion with edema peripheral. Another patient shows hyponatremia and hypochloremia. Other patients except those above show single symptoms.

**TABLE 2:** Adverse drug reactions.^b^

	Patients, *n* (%)
Patients in safety analysis set	1049 (100)
ADR	259 (24.7)
Serious ADR	7 (0.7)

Type of ADR	Proportion with ADR, *n* (%)	
	Overall	Serious
Infections and infestations	2 (0.2)	
Gastroenteritis^a^	2 (0.2)	
Metabolism and nutrition disorders	144 (13.7)	4 (0.4)
Hyperkalemia^a^	1 (0.1)	
Hypernatremia^a^	1 (0.1)	
Hypochloremia^a^	6 (0.6)	1 (0.1)
Hypokalemia^a^	1 (0.1)	
Hyponatremia	140 (13.3)	4 (0.4)
Decreased appetite^a^	2 (0.2)	
Psychiatric disorders	3 (0.3)	
Dysphoria^a^	1 (0.1)	
Insomnia^a^	2 (0.2)	
Nervous system disorders	28 (2.7)	
Dizziness	9 (0.9)	
Head discomfort^a^	1 (0.1)	
Headache	19 (1.8)	
Eye disorders	1 (0.1)	
Eyelid edema	1 (0.1)	
Ear and labyrinth disorders	1 (0.1)	
Vertigo^a^	1 (0.1)	
Cardiac disorders	3 (0.3)	1 (0.1)
Cardiac failure^a^	1 (0.1)	
Cardiac failure congestive	1 (0.1)	1 (0.1)
Palpitations^a^	1 (0.1)	
Vascular disorders	5 (0.5)	1 (0.1)
Blood pressure fluctuation^a^	1 (0.1)	
Hypertension^a^	4 (0.4)	1 (0.1)
Respiratory, thoracic and mediastinal disorders	4 (0.4)	1 (0.1)
Asthma^a^	1 (0.1)	
Dyspnoea^a^	1 (0.1)	
Orthopnea^a^	1 (0.1)	
Pleural effusion^a^	1 (0.1)	1 (0.1)
Gastrointestinal disorders	29 (2.8)	
Abdominal discomfort	3 (0.3)	
Abdominal pain^a^	1 (0.1)	
Abdominal pain upper^a^	1 (0.1)	
Constipation	3 (0.3)	
Diarrhea	6 (0.6)	
Dry mouth^a^	1 (0.1)	
Dry mouth	2 (0.2)	
Nausea	11 (1.1)	
Vomiting	3 (0.3)	
Feces soft^a^	1 (0.1)	
Hepatobiliary disorders	2 (0.2)	1 (0.1)
Cholecystitis acute^a^	1 (0.1)	1 (0.1)
Hepatic function abnormal	1 (0.1)	
Skin and subcutaneous tissue disorders	9 (0.9)	
Dermatitis^a^	1 (0.1)	
Eczema	1 (0.1)	
Erythema^a^	1 (0.1)	
Hyperhidrosis^a^	1 (0.1)	
Pruritus	5 (0.5)	
Rash	2 (0.2)	
Musculoskeletal and connective tissue disorders	2 (0.2)	
Rheumatoid arthritis^a^	1 (0.1)	
Musculoskeletal stiffness^a^	1 (0.1)	
Renal and urinary disorders	13 (1.2)	
Dysuria	3 (0.3)	
Nocturia	3 (0.3)	
Pollakiuria	3 (0.3)	
Renal impairment	4 (0.4)	
General disorders and administration site conditions	52 (5.0)	1 (0.1)
Condition aggravated^a^	1 (0.1)	
Face edema	1 (0.1)	
Fatigue	1 (0.1)	
Feeling abnormal	1 (0.1)	
Generalized edema	1 (0.1)	
Malaise	22 (2.1)	
Edema	2 (0.2)	
Edema peripheral	18 (1.7)	1 (0.1)
Thirst^a^	5 (0.5)	
Symptom recurrence	1 (0.1)	
Investigations	18 (1.7)	1 (0.1)
Glomerular filtration rate decreased^a^	2 (0.2)	1 (0.1)
Weight decreased^a^	1 (0.1)	
Weight increased^a^	3 (0.3)	
White blood cell count increased^a^	1 (0.1)	
Brain natriuretic peptide increased	6 (0.6)	
Urine output decreased^a^	1 (0.1)	
N‐terminal prohormone brain natriuretic peptide increased^a^	7 (0.7)	

Abbreviation: ADR, adverse drug reaction.

^a^ ADRs not predicted from package inserts.

^b^ Japanese MedDRA version 26.0.

### 3.5 Hyponatremia

Hyponatremia was the most common ADR, with 155 events in 140 patients, representing 13.3% of the safety analysis set (Table 2). The diagnosis of hyponatremia was determined at the discretion of the attending physicians and therefore did not necessarily correlate with the measured serum sodium concentrations. Notably, 4 patients were reported as hyponatremia despite having serum sodium levels > 135 mEq/L, whereas 13 patients with serum sodium levels < 135 mEq/L were not reported as hyponatremia.

The serum sodium level was measured in 871 patients (83.0%) before treatment, in 748 patients (71.3%) at 1 week, 745 patients (71.8%) at 4 weeks, 420 patients (45.2%) at 12 weeks, 338 patients (45.6%) at 24 weeks, and 270 patients (50.8%) at 52 weeks after treatment. Although the Japanese labeling states that “…The treatment should be discontinued if serum sodium level is below 135 mEq/L as reference or rapid decrease,” desmopressin ODT was administered to 10 patients with a pre‐treatment serum sodium level of < 135 mmol/L, 6 of whom developed hyponatremia (Table 3). Among 871 patients whose serum sodium levels were measured before treatment, 861 patients had levels > 135 mmol/L. Post‐treatment hyponatremia (serum sodium < 135 mmol/L) occurred more frequently in patients whose baseline serum sodium was between 135 and < 140 mmol/L (26.7%, 66/247 patients) compared with those whose levels were between 140 and < 148 mmol/L (9.2%, 52/568 patients).

**TABLE 3:** Relationship between the nadir serum sodium level after administration of desmopressin and the baseline serum sodium level.

Serum sodium level before administration of desmopressin (mmol/L)	Nadir serum sodium level after administration of desmopressin (mmol/L)														
	< 126			126 to < 130			130 to < 135			≥ 135			NM		
	Participants, *n*	ADR reported as hyponatremia		Participants, *n*	ADR reported as hyponatremia		Participants, *n*	ADR reported as hyponatremia		Participants, *n*	ADR reported as hyponatremia		Participants, *n*	ADR reported as hyponatremia	
		Overall	Serious		Overall	Serious		Overall	Serious		Overall	Serious		Overall	Serious
< 126	0	0	0	1	1	0	0	0	0	0	0	0	0	0	0
126 to < 130	0	0	0	0	0	0	0	0	0	1	0	0	0	0	0
130 to < 135	1	1	0	2	2	0	4	2	0	0	0	0	1	0	0
135 to < 140	8	8	2	15	15	0	43	35	0	181	2	0	9	0	0
140 to < 148	3	3	1	9	9	0	40	37	0	516	2	0	34	0	0
≥ 148	0	0	0	0	0	0	0	0	0	3	0	0	0	0	0
NM	2	2	0	5	5	0	16	16	1	125	0	0	30	0	0
Total	14	14	3	32	32	0	103	90	1	826	4	0	74	0	0

Abbreviations: ADR, adverse drug reaction; NM, not measured.

In the overall analysis population (*N* = 1049), hyponatremia occurred most frequently during the period of 1 to < 2 weeks affecting 71 patients (6.8%, 71/1049), followed by the period of 4 to < 12 weeks, with 22 patients affected (2.1%, 22/1049). Among the 140 patients who developed hyponatremia, these periods accounted for 50.7% (71/140) and 15.7% (22/140), respectively. Of those who developed hyponatremia whose pre‐serum sodium levels were between 135 and < 140 mmol/L, 36 (60.0%, 36/60) and 7 (11.7%, 7/60) patients reported hyponatremia in 1 to < 2 weeks and 4 to < 12 weeks after initiating treatment, respectively. Among patients whose pre‐serum sodium levels were between 140 and < 148 mmol/L, 21 (41.2%, 21/51) and 11 (21.6%, 11/51) patients reported hyponatremia in 1 to < 2 weeks and 4 to < 12 weeks after initiating treatment, respectively. In total, 26 patients of hyponatremia were observed after 12 weeks of desmopressin administration (Table 4).

**TABLE 4:** Relationship between the baseline serum sodium level and time to onset of hyponatremia.

Serum sodium level before administration of desmopressin (mmol/L)	1 day	2 days to < 1 week	1 to < 2 weeks	2 to < 4 weeks	4 to < 12 weeks	12 to < 26 weeks	26 to < 52 weeks	≥ 52 weeks	Unknown
< 126	0	0	0	0	1	0	0	0	0
126 to < 130	0	0	0	0	0	0	0	0	0
130 to < 135	0	0	3	0	2	0	0	0	0
135 to < 140	0	5	36	5	7	3	3	0	1
140 to < 148	0	1	21	2	11	8	7	0	1
≥ 148	0	0	0	0	0	0	0	0	0
NM	0	1	11	5	1	1	2	2	0
Total	0	7	71	12	22	12	12	2	2

Abbreviation: NM, not measured.

**FIGURE 3:**
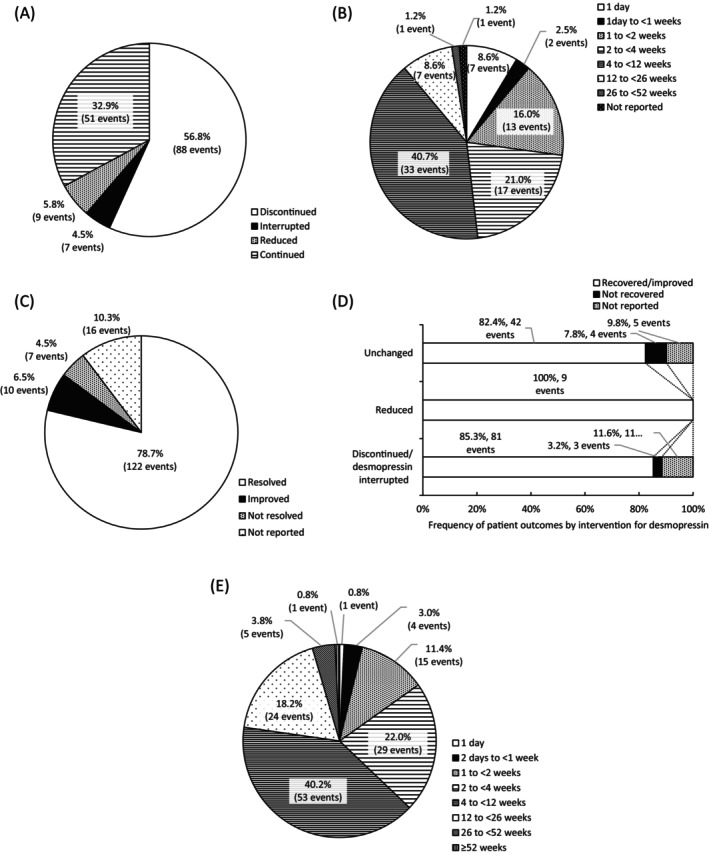
Response to hyponatremia and subsequent patient outcomes. (A) Response following the onset of hyponatremia after administration of desmopressin for up to 12 weeks. (B) Time to recover of hyponatremia in patients who developed hyponatremia from desmopressin administration to 12 weeks. (C) Outcomes of hyponatremia event. (D) Frequency of patient outcomes by interventions for desmopressin. (E) Time from discontinuation or termination of desmopressin administration to recovery or resolution of symptoms.

After hyponatremia development, desmopressin treatment was discontinued or interrupted in 95 events (61.3%, 95/155) and the dose was reduced in 9 events (6.0%, 9/155) (Figure 3A). Of the 95 events where desmopressin was discontinued or interrupted due to hyponatremia, recovery was achieved in 81 events (85.3%); the remaining 11 events (11.6%) had unknown outcomes, and 3 events (3.2%) were unrecovered (Figure 3D). The time to recover in those 81 events was as follows: 1 day in 7 events (8.6%), 1 to < 2 weeks in 13 events (16.0%), 2 to < 4 weeks in 17 events (21.0%), and 4 to < 12 weeks in 33 events (40.7%) (Figure 3B). Among 155 hyponatremia events, physicians reported that the hyponatremia resolved in 122 events (78.8%), improved in 10 events (6.5%), and did not resolve in 7 events (4.5%), while the outcomes for the remaining 16 events (10.3%) were not reported (Figure 3C). All 9 events where the desmopressin dose was reduced resulted in either resolution or improvement. Of the 51 events with no dose change, 42 events (82.4%) resolved or improved, 4 events (7.8%) did not resolve, and 5 events (9.8%) had unreported outcomes. Among the 132 events where hyponatremia resolved or improved, hyponatremia was resolved or improved in 1 to < 2 weeks in 15 events (11.4%), 2 to < 4 weeks in 29 events (22.0%), 4 to < 12 weeks in 53 events (40.2%), and 12 to < 26 weeks in 24 events (18.2%) (Figure 3E).

Although fewer patients were initiated on a 25 μg dose (493/1049, 47.0%) compared to those who started on a 50 μg dose (556/1049, 53.0%), the incidence of hyponatremia was higher in the 25 μg group (81/493, 16.4%) than in the 50 μg group (59/556, 10.6%). Notably, among the 81 patients who developed hyponatremia and had initiated treatment with a desmopressin ODT dose of 25 μg, only 10 patients had their dose increased to 50 μg. The remaining 71 patients developed hyponatremia while continuing on the initial 25 μg dose. (Table 5). An analysis of the timing of hyponatremia onset stratified by initial desmopressin dose revealed that the majority of patients developed hyponatremia between weeks 1 and 4. In the group that initiated treatment with 25 μg, 39 patients (39/493, 7.9%) of hyponatremia were observed during this period, compared with 26 patients (26/556, 4.6%) in the group that started with 50 μg (Table 6).

**TABLE 5:** Dose of desmopressin at the start of treatment and on the day before onset of hyponatremia.

	Initial dose of desmopressin, *n* (%)	Dose on the day before diagnosis of hyponatremia		Patients without hyponatremia, *n* (%)
		25 μg, *n* (%)	50 μg, *n* (%)	
25 μg	493 (47.0)	71 (6.7)	10 (1.0)	412 (39.3)
50 μg	556 (53.0)	2 (0.2)	57 (5.4)	497 (47.4)

*Note:* Denominator for % is *N*: Represents the safety analysis set (1049 patients).

**TABLE 6:** Interval between the initial dose of desmopressin and first detection of a serum sodium level < 135 mmol/L (ADR reported as hyponatremia).

Time of assessment	Initial dose of desmopressin			
	25 μg		50 μg	
	Participants, *n*	Developed hyponatremia (%)	Participants, *n*	Developed hyponatremia (%)
Within 1 week	493	39 (7.9)	556	26 (4.6)
Within 4 weeks	485	15 (3.1)	552	22 (4.0)
Within 12 weeks	436	8 (1.8)	493	3 (0.6)
Within 24 weeks	356	5 (1.4)	385	2 (0.5)
Within 52 weeks	262	11 (4.2)	270	5 (1.9)
Unclear		3		1

A comparison of patient background at the initial dose of 25 and 50 μg showed differences in age, body weight, Ccr estimate by Cockcroft–Gault equation before desmopressin, benign prostatic hyperplasia, overactive bladder, on medications for overactive bladder and concomitant medications for nocturia (including BPH and OAB) (see Table 1).

**FIGURE 4:**
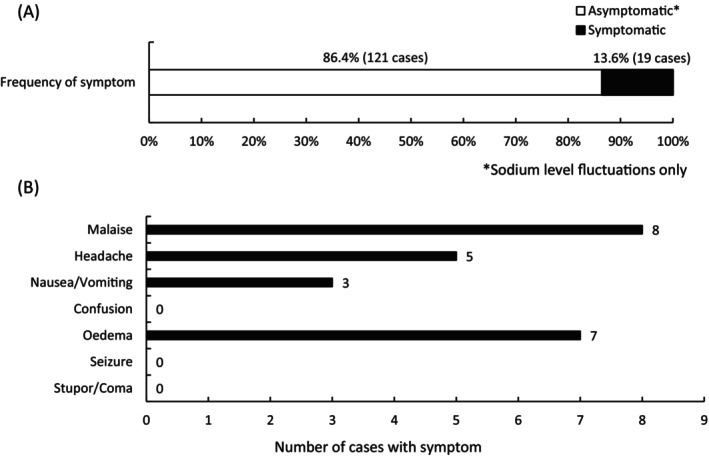
(A) Frequency of hyponatremia patients stratified by presence or absence of symptoms. (B) Details of symptoms associated with hyponatremia.

Hyponatremia‐associated symptoms were observed in 19 (13.6%) of the 140 patients (Figure 4A). The reported symptoms included malaise in 8 patients, edema in 7, headache in 5, and nausea and/or vomiting in 3; however, no patients of confusion, seizure, stupor, or coma were reported (Figure 4B). Multiple symptoms were observed in 4 patients: fatigue and headache in 2 patients, nausea and/or vomiting and edema in 1 patient, fatigue and edema in 1 patient. No patients with acute diseases that may develop hyponatremia, such as systemic infection, fever, gastroenteritis, diarrhea, were identified.

### 3.6 Association of Patient Characteristics With Desmopressin‐Induced Hyponatremia

Statistically significant differences in the incidence of hyponatremia were observed with the predefined categories in age, body weight, BMI, Ccr estimated by Cockcroft–Gault equation before desmopressin, hemoglobin level before desmopressin, past medical history, history of BPH, history of QAB, initial dose of desmopressin, serum sodium level before desmopressin, proportion of monocytes in differential WBC count before desmopressin, blood urea nitrogen level before desmopressin (Table 7).

**TABLE 7:** Association of patient characteristics with desmopressin‐induced hyponatremia.

Characteristics^a^		Participants, *n*	Hyponatremia (%)	*p* ^b^
Age (years)	< 75	420	28 (6.7)	< 0.001
	≥ 75	629	112 (17.8)	
Body weight (kg)	< 40	3	0 (0)	< 0.001
	40 to < 50	19	8 (42.1)	
	50 to < 60	147	31 (21.1)	
	60 to < 70	209	20 (9.6)	
	70 to < 80	109	9 (8.3)	
	≥ 80	31	1 (3.2)	
	Unknown	531	71 (13.4)	
BMI (kg/m^2^)	< 18.5	29	8 (27.6)	0.017
	18.5 to < 25	344	51 (14.8)	
	25 to < 30	116	8 (6.9)	
	≥ 30	14	1 (7.1)	
	Unknown	546	72 (13.2)	
Ccr estimated by Cockcroft–Gault equation before desmopressin (mL/min)	< 30	2	2 (100)	< 0.001
	30 to < 50	77	20 (26.0)	
	50 to < 80	242	25 (10.3)	
	≥ 80	103	8 (7.8)	
	NM	625	85 (13.6)	
Hemoglobin level before desmopressin (g/dL)	< 12.1	104	19 (18.3)	< 0.001
	12.1 to < 13.1	146	37 (25.3)	
	13.1 to < 16.3	504	58 (11.5)	
	16.3 to < 18.0	38	2 (5.3)	
	≥ 18.0	2	0 (0)	
	NM	255	24 (9.4)	
Past medical history	No	862	102 (11.8)	0.003
	Yes	187	38 (20.3)	
History of BPH	No	1018	129 (12.7)	0.001
	Yes	31	11 (35.5)	
History of OAB	No	1038	136 (13.1)	0.047
	Yes	11	4 (36.4)	
Initial dose of desmopressin (μg)	25	493	81 (16.4)	0.006
	50	556	59 (10.6)	
Night‐time urine volume before desmopressin	< 200	0	‐ (−)	0.013
	200 to < 300	2	0 (0)	
	300 to < 400	15	1 (6.7)	
	400 to < 500	29	0 (0)	
	500 to < 600	64	6 (9.4)	
	600 to < 700	110	18 (16.4)	
	700 to < 800	108	7 (6.5)	
	800 to < 900	135	19 (14.1)	
	900 to < 1000	89	19 (21.4)	
	≥ 1000	219	40 (18.3)	
	NM	278	30 (10.8)	
Serum sodium level before desmopressin (mmol/L)	< 126	1	1 (100)	< 0.001
	126 to < 130	1	0 (0)	
	130 to < 135	8	5 (62.5)	
	135 to < 140	256	60 (23.4)	
	140 to < 148	602	51 (8.5)	
	≥ 148	3	0 (0)	
	NM	178	23 (12.9)	
Proportion of monocytes in differential WBC count before desmopressin (%)	< 2.0	1	0 (0)	< 0.001
	2.0 to < 10.0	196	23 (11.7)	
	≥ 10.0	15	8 (53.3)	
	NM	837	109 (13.0)	
Blood urea nitrogen level before desmopressin (mg/dL)	< 8.0	4	2 (50.0)	0.017
	8.0 to < 22.0	586	72 (12.3)	
	≥ 22.0	110	21 (19.1)	
	Unknown	349	45 (12.9)	

Abbreviations: BMI, body mass index; BPH, benign prostatic hyperplasia; Ccr, creatinine clearance; NM, not measured; OAB, overactive bladder; WBC, white blood cell.

^a^ Only patient characteristics showing a significant difference in the incidence of hyponatremia between each group are shown.

^b^ Fisher's exact test (two‐tailed) for two categories, Chi‐squared test (two‐tailed) for three or more categories.

Among the variables analyzed shown in Table 7, only age, history of BPH, and serum sodium level before desmopressin were identified as significant factors associated with the development of hyponatremia with multi‐variable analysis. Patients aged ≥ 75 years had a significantly higher incidence of hyponatremia compared with those aged < 75 years (OR 2.681, 95% CI 1.596–4.505). Similarly, patients with a history of BPH had a higher incidence of hyponatremia compared with those without the history (OR 2.945, 95% CI 1.053–8.233). In contrast, patients with a baseline serum sodium level of 140 to < 148 mmol/L had a significantly lower incidence of hyponatremia compared with those whose baseline level was 135 to < 140 mmol/L (OR 0.297, 95% CI 0.183–0.482) (Table 8).

**TABLE 8:** Multivariate logistic regression analysis of factors affecting the occurrence of hyponatremia.

Characteristics		Participants, *n*	Hyponatremia (%)	OR	95% CI for OR	*p* ^a^
Age (years)	< 75	420	28 (6.7)	Reference		< 0.001
	≥ 75	629	112 (17.8)	2.681	1.596–4.505	
History of BPH	No	1018	129 (12.7)	Reference		0.040
	Yes	31	11 (35.5)	2.945	1.053–8.233	
Serum sodium level before desmopressin (mmol/L)	< 126	1	1 (100)	> 999.999	< 0.001 to > 999.999	< 0.001
	126 to < 130	1	0 (0)	< 0.001	< 0.001 to > 999.999	
	130 to < 135	8	5 (62.5)	5.256	1.052–26.269	
	135 to < 140	256	60 (23.4)	Reference		
	140 to < 148	602	51 (8.5)	0.297	0.183–0.482	
	≥ 148	3	0 (0)	< 0.001	< 0.001 to > 999.999	
	NM	178	23 (12.9)	0.657	0.271–1.593	

*Note:* The relevance between the predicted probability and the response of the observed data: 0.823. Comprehensive *p*‐value in multivariate logistic regression: 0.002.

Abbreviations: BPH, benign prostatic hyperplasia; CI, confidence interval; NM, not measured; OR, odds ratio.

^a^ Wald test.

## 4 Discussion

This PMS presents the largest cohort of patients treated with desmopressin ODT for nocturia due to NP based on real‐world clinical data. Among the 1049 patients included in the safety analysis set, ADRs were reported in 259 patients (24.7%), with hyponatremia being the most frequently reported ADR, occurring in 140 patients (13.3%). These results are consistent with other reports [12, 13] indicating that desmopressin ODT is associated with a risk of hyponatremia.

Patients with nocturia are often elderly, and advanced age is a known risk factor for hyponatremia [9]. Since 60% of the patients (629/1049) were aged ≥ 75 years in the present survey, the total safety analysis set was a high risk population of hyponatremia. In the present study, the incidence of Hyponatremia was higher in patients who initiated and maintained treatment with a low dose of 25 μg desmopressin compared to those who started and remained on a 50 μg dose. The selection and adjustment of desmopressin dosage were at the discretion of the attending physicians, and therefore, a potential dose selection bias, that is, prescription of low dose desmopressin to elderly or high‐risk patients, cannot be excluded. Therefore, since the baseline characteristics differed between the group that received an initial dose of 25 μg and the group that received 50 μg (see Table 1), the incidence of hyponatremia cannot be directly compared between the two groups. In Japan, physicians carefully administer 25 μg to patients at risk. These findings suggest that even when initiating treatment with a low dose of 25 μg, close monitoring of hyponatremia is required.

Hyponatremia is known to be difficult to detect based solely on clinical symptoms [14]. In the present survey, among the 140 patients who developed hyponatremia, only 19 patients demonstrated hyponatremia symptoms, while the remaining 121 patients (86.4%) were asymptomatic. The initial decline in serum sodium levels to below 135 mmol/L was most commonly observed within 1 week of desmopressin initiation, regardless of the starting dose. However, 26 patients developed hyponatremia more than 12 weeks after treatment onset. These findings highlight the significance of serum sodium monitoring throughout the course of desmopressin therapy to enable early detection of hyponatremia.

Among the 155 events in which hyponatremia occurred, treatment was discontinued or suspended in 95 events, and recovery or improvement was observed in 132 events. Although it depends on the timing of measurement, most patients of desmopressin‐induced hyponatremia resolved or improved within 1 month of cessation or suspension of treatment. Most events of hyponatremia are reversible and resolve following discontinuation of treatment; however, recovery should be assessed based on serum sodium levels rather than clinical symptoms. Based on these findings, it is important to follow the statement in the Japanese labeling that “Serum sodium should be measured within the first week (3–7 days) after initiation of the treatment or increasing the dose, and at the first 1 month of the treatment and regularly after 1 month. The treatment should be discontinued if serum sodium level is below 135 mEq/L as reference or rapid decrease.”

In this survey, the following were identified as risk factors for desmopressin‐induced hyponatremia: age ≥ 75 years, history of BPH, and a low serum sodium level before desmopressin. To date, no research reported an association between BPH and hyponatremia. Although only 31 patients in this survey had a history of BPH, the proportion was high at 74.2% in those aged ≥ 75 years. Given the small sample size of patients with a history of benign prostatic hyperplasia and the wide confidence interval (1.053–8.233), it is difficult to regard these findings as indicating an equivalent risk level as advanced age and lower baseline serum sodium levels prior to desmopressin treatment, and the clinical significance appears limited. The Japanese labeling states that desmopressin ODT is contraindicated in “patients with moderate and severe renal insufficiency (creatinine clearance below 50 mL/min),” and it has been recommended that patients with impaired renal function should avoid using desmopressin because of the high likelihood of SAEs [15].

These findings indicate that desmopressin ODT should be started at a low dose with monitoring of the serum sodium level frequently in patients aged ≥ 75 years, those with underlying conditions such as BPH or impaired renal function, and those with a serum sodium level of < 140 mmol/L, even if the level is within the reference range.

This survey has some limitations. First, the survey implies patient selection bias and recall bias. It was conducted as a PMS collecting real‐world clinical data. Consequently, attending physicians managed patients with diverse backgrounds at their discretion, and prescribed high‐risk patients for hyponatremia with lower dose, 25 μg. As the survey is designed to enroll patients within 1 week after starting their treatment, patients who developed hyponatremia at an early stage may be excluded for the enrollment. Second, the diagnosis of hyponatremia was not made by serum sodium level but by decisions based on attending doctors. There were instances in which hyponatremia was diagnosed despite serum sodium levels not falling below 135 mmol/L following desmopressin administration, and conversely, patients in whom hyponatremia was not diagnosed even when serum sodium levels were below 135 mmol/L. These discrepancies may have resulted in either an underestimation or overestimation of the incidence of hyponatremia.

## 5 Conclusions

This PMS represents the largest investigation to date of the incidence of ADRs associated with desmopressin ODT for the treatment of male nocturia due to NP. Hyponatremia was identified as the most frequently observed ADR. Advanced age (≥ 75 years) and lower baseline serum sodium levels prior to desmopressin treatment were identified as *significant* risk factors for hyponatremia.

Most hyponatremia events associated with desmopressin treatment emerged within 1–2 weeks of treatment initiation, although later onset after 12 weeks was also observed. Hyponatremia was generally reversible following discontinuation of desmopressin treatment. However, it was often asymptomatic and occurred even at the low dose of 25 μg. These findings underscore the importance of appropriate monitoring of serum sodium levels, with careful consideration of individual risk factors.

## Funding

This work was supported by Post‐Marketing Surveillance, Medical Affairs, Ferring Pharmaceuticals Co. Ltd., Tokyo, Japan.

## Ethics Statement

This survey was conducted in accordance with the Ministerial Ordinance on Good Post‐Marketing Study Practice (Ministry of Health, Labour and Welfare Ordinance No. 171, December 20, 2004).

## Consent

According to good post‐marketing study practice in Japan, informed consent was not required for this post‐marketing survey. However, informed consent was obtained from patients to participate in the survey and for publication of the data collected.

## Conflicts of Interest

In this PMS, analysis and publication were funded by Ferring Pharma Inc. Yoshimasa Ogawa and Atsushi Nakano are employees of Ferring Pharmaceuticals Co. Ltd. Kiyotoshi Kuramoto is an employee of Kissei Pharmaceutical Co. Ltd.

## Supporting information


**File S1:** CRF1_From the start of administration of this drug to 12 weeks later.

**File S2:** CRF2_Data for 13–52 weeks after the start of administration of this drug.

## Data Availability

The data sets generated and/or analyzed during the current survey are available from the corresponding author upon reasonable request.
