# Supplementary material for: Long‐Term Safety of Desmopressin Orally Disintegrating Tablets in Men With Nocturia due to Nocturnal Polyuria: Final Results of a Specified Drug Use‐Results Survey in Japan
Source: Low Urin Tract Symptoms. 2026 Mar 17;18(2):e70052. doi: 10.1111/luts.70052 (PMC12995508; doi:10.1111/luts.70052)
Supplement: Supplementary file 1 — File S1: CRF1_From the start of administration of this drug to 12 weeks later. [file LUTS-18-e70052-s002.pdf]

≪CRF 1 'From the start of administration of this drug to 12 weeks later' for survey data (total 13 pages)≫

【 patient characteristics 】 -Patient identification

・Please enter the patient background at the start of administration of this drug.
・Please measure height and weight as much as possible, as they are listed as risk factors for hyponatremia.

Patient's initials※1	Last name	First name	Sex	■ Male	Date of birth or age※2 (at the start of administration of this drug)	□ Taisho □ Showa □ Heisei □ Gregorian calendar<br>Year Month Day<br>Years	patient to identify No.	( )
Height	(. cm) □ Unknown				Weight	(. kg) □ Unknown	Medical care category	□ Outpatient □ Inpatient

※1 If you cannot provide the patient's initials, please enter XX.
※2 From a privacy protection standpoint, if the full date of birth cannot be disclosed, please enter up to 'year, month' or the 'age' at the start of administration of this drug.

【 patient characteristics 】 -Patient condition

・Please enter the reason for use of this drug.

□ Indication	□ Other than indication (disease name:)
nocturia from nocturnal polyuria in men	

・Please enter the clinical test results before the start of administration of this drug.
・Please measure as much as possible, as it is listed as a risk factor for hyponatremia.

Creatinine Clearance	________ mL/min (□2 hours □24 hours)<br>(Measurement date: Year Month Day)<br>□ Not measured	Hemoglobin	________ g/dL<br>(Measurement date: Year Month Day)<br>□ Not measured

・Please enter the patient condition before the start of administration of this drug.

Smoking history	□ No □ Yes □ Past smoking experience □ Unknown<br>→ Smoking years: about ( ) years 1 day number: about ( ) cigarettes/day
Alcohol consumption history	□ Daily □ Occasionally □ Hardly drink (cannot drink) □ Unknown<br>→ Daily alcohol intake on drinking days:<br>□ Less than 1 go □ 1 go to less than 2 go □ 2 go to less than 3 go □ 3 go or more
PS (Performance Status)	□ 0: Able to perform activities without any problems. Able to perform daily life as before the onset without restrictions.<br>□ 1: Physically strenuous activities are restricted, but walking is possible, and light work or sitting work can be performed.<br>□ 2: Walking is possible and all personal care is possible, but work cannot be done. Spend more than 50% of the day out of bed.<br>□ 3: Can only perform limited personal care. Spend more than 50% of the day in bed or a chair.<br>□ 4: Completely unable to move. Completely unable to perform personal care. Spend completely in bed or a chair.

【 patient characteristics 】 - Medical history

・Please enter the presence or absence of renal-related diseases at the start of administration of this drug.
・If renal impairment is 'ongoing' or 'cured,' please also enter the severity of the disease.

disease name	which way the wind blows			Severity Measures
Renal impairment	□ No medical history	□ Ongoing	□ Cured	□ Mild □ Moderate □ Severe
Benign Prostatic Hyperplasia	□ No medical history	□ Ongoing	□ Cured	
overactive bladder	□ No medical history	□ Ongoing	□ Cured	
interstitial bladder cystitis	□ No medical history	□ Ongoing	□ Cured	

・Please enter the presence or absence of a history of arthritis, as it is listed as a risk factor for hyponatremia.
・Please enter the presence or absence of a history of hypertension, obesity, diabetes, valvular disease, cardiomyopathy, and coronary artery disease, which are considered risk factors for heart failure.

disease name	which way the wind blows			disease name	which way the wind blows		
Arthritis	□ No medical history	□ Ongoing	□ Cured	Hypertension	□ No medical history	□ Ongoing	□ Cured
OBESITY	□ No medical history	□ Ongoing	□ Cured	Diabetes mellitus	□ No medical history	□ Ongoing	□ Cured
valvular disease	□ No medical history	□ Ongoing	□ Cured	Cardiomyopathy	□ No medical history	□ Ongoing	□ Cured
Coronary artery disease	□ No medical history	□ Ongoing	□ Cured				

・Please enter the medical history at the start of administration of this drug, excluding the above renal impairment, benign prostatic hyperplasia, overactive bladder, interstitial bladder cystitis, arthritis, hypertension, obesity, diabetes, valvular disease, cardiomyopathy, and coronary artery disease.
・Please enter all medical history at the start of administration of this drug, including diseases and symptoms (such as liver disease) that may cause hyponatremia other than the above.
・Please enter the medical history at the start of administration of this drug, including allergic reactions and diseases (sensitization to foreign substances such as pollen, food, and drugs).

□ Yes	□ No

No.	disease name	which way the wind blows	No.	disease name	which way the wind blows
1		□ Ongoing □ Cured	9		□ Ongoing □ Cured
2		□ Ongoing □ Cured	10		□ Ongoing □ Cured
3		□ Ongoing □ Cured	11		□ Ongoing □ Cured
4		□ Ongoing □ Cured	12		□ Ongoing □ Cured
5		□ Ongoing □ Cured	13		□ Ongoing □ Cured
6		□ Ongoing □ Cured	14		□ Ongoing □ Cured
7		□ Ongoing □ Cured	15		□ Ongoing □ Cured
8		□ Ongoing □ Cured	18		□ Ongoing □ Cured
9		□ Ongoing □ Cured	19		□ Ongoing □ Cured
10		□ Ongoing □ Cured	20		□ Ongoing □ Cured

Suppleme nt : Disease condition: 'Complication' refers to a disease present at the start of administration of this drug, and 'past history' refers to a disease that was present before the start of administration of this drug but is cured at the start of administration.

【 records on dosing 】 - Use of this drug

- Please describe the usage status after the administration of this product (during the observation period in this survey form).
- If the dosage and administration are changed, please enter separately in another row. If treatment is continued with the same dosage and administration, do not enter the 'end date' and check 'ongoing'.
- If a new administration occurs due to dose modification or drug holiday, please enter the reason for dose modification or drug holiday. It is not necessary for ongoing rows.

No.	Daily dose	Treatment period (from the start of administration to the end of administration or the date of dosage and administration change)			Reason for dose modification
1	□ 25µg<br>□ 50µg<br>□ ( )µg	year month day	～	year month day<br>/ □ Continued	AE(No)<br>□ reasons other than adverse events
2	□ 25µg<br>□ 50µg<br>□ ( )µg	year month day	～	year month day<br>/ □ Continued	AE(No)<br>□ reasons other than adverse events
3	□ 25µg<br>□ 50µg<br>□ ( )µg	year month day	～	year month day<br>/ □ Continued	AE(No)<br>□ reasons other than adverse events
4	□ 25µg<br>□ 50µg<br>□ ( )µg	year month day	～	year month day<br>/ □ Continued	AE(No)<br>□ reasons other than adverse events
5	□ 25µg<br>□ 50µg<br>□ ( )µg	year month day	～	year month day<br>/ □ Continued	AE(No)<br>□ reasons other than adverse events
6	□ 25µg<br>□ 50µg<br>□ ( )µg	year month day	～	year month day<br>/ □ Continued	AE(No)<br>□ reasons other than adverse events
7	□ 25µg<br>□ 50µg<br>□ ( )µg	year month day	～	year month day<br>/ □ Continued	AE(No)<br>□ reasons other than adverse events
8	□ 25µg<br>□ 50µg<br>□ ( )µg	year month day	～	year month day<br>/ □ Continued	

Supplement: If the reason for dosage and administration change or drug holiday is 'Adverse Events', please enter details in 【Safety Evaluation】 (pages 9 - 11).

【Status of administration of this product at the end of the observation period】 - Patient outcomes

・Please enter the medical classification, administration status of this product, and progress information at the end of the observation period in this survey form.

date confirmed	(year month day)
Medical classification	□ Hospitalization※ □ Outpatient □ Unknown<br>※If the reason for hospitalization is 'Occurrence and treatment of Adverse Events', please enter details in 【Safety Evaluation】 (pages 9 - 11).
Use of secukinumab	□ Ongoing
	□ Terminated (End date: year month day)
	□ Discontinued (Discontinuation date: year month day)<br>〔Reason for discontinuation〕 □ Patient's wish (for reasons other than adverse events)<br>※Select one □ Adverse Events ⇒ Please enter details in 【Safety Evaluation】 (pages 9 - 11). 〔Adverse Events No.: 〕<br>Progression/worsening of primary disease / complications (including associated symptoms), death<br>【 Safety assessment 】 - Please record on the Adverse Event(s) page.<br>□ Lack of efficacy<br>□ Transfer (Transfer date: year month day)<br>□ Did not visit (Last visit date: year month day)<br>□ Other (Details:)

Supplement: 'Termination' refers to the end of treatment with this product due to improvement (such as significant efficacy), while 'Discontinuation' refers to the unavoidable cessation of treatment with this product at the medical institution due to progression/worsening of the primary disease, occurrence of adverse events, patient's wish, death, transfer, etc.

【 Combination Therapy 】 - Treatment drugs for nocturia other than this product

・Please enter the treatment drugs for nocturia other than this product (drug therapy) during the observation period in this survey form. If the dosage and administration are changed, please enter separately in another row. In the case of as-needed medication, please enter the single dose in the daily dose column.
・If treatment is continued with the same dosage and administration, do not enter the 'end date' and check 'ongoing'.

□ Yes	□ No

No.	Name of drug	Daily dose (units)	Route of administration	As-needed	Treatment period (from the start of administration to the end of administration or change)
1			□ PO<br>□ Injection<br>□ Topical product<br>□ ( )	□	Start date: year month day / □ Before administration of this product<br>End date: year month day / □ Ongoing
2			□ PO<br>□ Injection<br>□ Topical product<br>□ ( )	□	Start date: year month day / □ Before administration of this product<br>End date: year month day / □ Ongoing
3			□ PO<br>□ Injection<br>□ Topical product<br>□ ( )	□	Start date: year month day / □ Before administration of this product<br>End date: year month day / □ Ongoing
4			□ PO<br>□ Injection<br>□ Topical product<br>□ ( )	□	Start date: year month day / □ Before administration of this product<br>End date: year month day / □ Ongoing
5			□ PO<br>□ Injection<br>□ Topical product<br>□ ( )	□	Start date: year month day / □ Before administration of this product<br>End date: year month day / □ Ongoing

【 Combination Therapy 】 - Combination therapy for nocturia

・Please enter the combination therapy for nocturia other than drug therapy during the observation period in this survey form.
・For drug treatment for nocturia, please enter in 【Combination Therapy】- Treatment drugs for nocturia other than this product (page 4).
・If the same treatment is continued at the end of the observation period in this survey form, do not enter the 'end date' and check 'ongoing'.

□ Yes	□ No

No.	Treatment	Testing period (from the start of treatment to the end of treatment)
1	□ Behavioral therapy<br>□ Exercise therapy<br>Other ( )	Start date: year month day / □ Before administration of this product<br>End date: year month day / □ Ongoing
2	□ Behavioral therapy<br>□ Exercise therapy<br>Other ( )	Start date: year month day / □ Before administration of this product<br>End date: year month day / □ Ongoing
3	□ Behavioral therapy<br>□ Exercise therapy<br>Other ( )	Start date: year month day / □ Before administration of this product<br>End date: year month day / □ Ongoing
4	□ Behavioral therapy<br>□ Exercise therapy<br>Other ( )	Start date: year month day / □ Before administration of this product<br>End date: year month day / □ Ongoing
5	□ Behavioral therapy<br>□ Exercise therapy<br>Other ( )	Start date: year month day / □ Before administration of this product<br>End date: year month day / □ Ongoing

【 test 】 - Clinical tests related to events that should be investigated intensively

・If the following test items were conducted before and during administration, please enter the clinical test results.
・Serum sodium levels must be measured before administration, at week 1, and at week 4 from the perspective of proper use.
・Regardless of the causal relationship with drug administration, if clinically undesirable abnormal fluctuations are determined, please enter in 【Safety Evaluation】 - Adverse Events (pages 9 - 11).

No.	Timepoint / Test item Unit		Baseline	during the drug administration			
			(4 weeks to 0 weeks before administration of this product) Week 4 (3 weeks to 6 weeks)	Week 1 (3 days to 10 days)	Week 4 (3 weeks to 6 weeks)	Week 12 (10 weeks to 14 weeks)	At discontinuation/termination
			year month day □ Not tested	2 year month day □ Not tested	year month day □ Not tested	year month day □ Not tested	year month day □ Not tested
1	serum creatinine	mg/dL					
2	White blood cell count	/μL					
3	Monocyte count/segment	□ /μL □ %					
4	serum sodium	mmol/L					
5	Urea nitrogen (BUN)	mg/dL					
6	Blood BNP	pg/mL					
7	NT-proBNP	pg/mL					

【 Combination Therapy 】 - Combination therapy for diseases other than the primary disease during the observation period

・Please enter the combination therapy (drug therapy and non-drug therapy) during the observation period in this survey form.
・For drug therapy, if the dosage and administration are changed, please enter separately in another row. In the case of as-needed medication, please enter the single dose in the daily dose column.
・If the same treatment (in the case of drug therapy, with the same dosage and administration) is continued, do not enter the 'end date' and check 'ongoing'.
・Please also enter the adverse event treatment drugs and adverse event treatment therapy.
・Please enter all information regarding drips and infusions.
・For drug treatment for nocturia, please enter in 【Combination Therapy】 - Treatment drugs for nocturia other than this product (page 4).
・For combination therapy for nocturia other than drug therapy, please enter in 【Combination Therapy】 - Combination therapy for nocturia (page 4).
・Since they are listed as risk factors for hyponatremia, if bone disease treatment drugs and hyperlipidemia treatment drugs are administered, please be sure to enter them.

≪ Drug therapy ≫

□ Yes	□ No

No.	Name of drug	Daily dose (units)	Route of administration	As-needed	Treatment period (from the start of administration to the end of administration or change)	Reasons for use ※Including associated symptoms
1			□ PO<br>□ Injection<br>□ Topical product<br>□ ( )	□	Start date: year month day<br>/ □ Before administration of this product<br>End date: year month day / □ Ongoing	□ Treatment of adverse events (No)<br>□ Treatment of complications (No)<br>□ Prevention
2			□ PO<br>□ Injection<br>□ Topical product<br>□ ( )	□	Start date: year month day<br>/ □ Before administration of this product<br>End date: year month day / □ Ongoing	□ Treatment of adverse events (No)<br>□ Treatment of complications (No)<br>□ Prevention

Supplement :
・If the reason for use is 'Treatment of adverse events', please enter details in 【Safety Evaluation】 - Adverse Events (pages 9 - 11).
・If it is 'Treatment of complications', please confirm that there is a corresponding description in 'Medical history' in 【Patient Background】 (page 2).
・Prevention includes, for example, gastrointestinal drugs prescribed for the prevention of gastritis.

(continued)

No.	Name of drug	Daily dose (units)	Route of administration	As-needed	Treatment period (from the start of administration to the end of administration or change)	Reasons for use ※Including associated symptoms
3			□ PO<br>□ Injection<br>□ Topical product<br>□ ( )	□	Start date: year month day<br>/ □ Before administration of this product<br>End date: year month day / □ Ongoing	□ Treatment of adverse events (No)<br>□ Treatment of complications (No)<br>□ Prevention
4			□ PO<br>□ Injection<br>□ Topical product<br>□ ( )	□	Start date: year month day<br>/ □ Before administration of this product<br>End date: year month day / □ Ongoing	□ Treatment of adverse events (No)<br>□ Treatment of complications (No)<br>□ Prevention
5			□ PO<br>□ Injection<br>□ Topical product<br>□ ( )	□	Start date: year month day<br>/ □ Before administration of this product<br>End date: year month day / □ Ongoing	□ Treatment of adverse events (No)<br>□ Treatment of complications (No)<br>□ Prevention
6			□ PO<br>□ Injection<br>□ Topical product<br>□ ( )	□	Start date: year month day<br>/ □ Before administration of this product<br>End date: year month day / □ Ongoing	□ Treatment of adverse events (No)<br>□ Treatment of complications (No)<br>□ Prevention
7			□ PO<br>□ Injection<br>□ Topical product<br>□ ( )	□	Start date: year month day<br>/ □ Before administration of this product<br>End date: Year Month Day / □ Continuation	□ Treatment of adverse events (No)<br>□ Treatment of complications (No)<br>□ Prevention
8			□ PO<br>□ Injection<br>□ Topical product<br>□ ( )	□	Start date: Year Month Day<br>/ □ From before administration of this drug<br>End date: Year Month Day / □ Continuation	□ Treatment of adverse events (No)<br>□ Treatment of complications (No)<br>□ Prevention
9			□ PO<br>□ Injection<br>□ Topical product<br>□ ( )	□	Start date: Year Month Day<br>/ □ From before administration of this drug<br>End date: 20 Year Month Day / □ Continuation	□ Treatment of adverse events (No)<br>□ Treatment of complications (No)<br>□ Prevention
10			□ PO<br>□ Injection<br>□ Topical product<br>□ ( )	□	Start date: Year Month Day<br>/ □ From before administration of this drug<br>End date: Year Month Day / □ Continuation	□ Treatment of adverse events (No)<br>□ Treatment of complications (No)<br>□ Prevention
11			□ PO<br>□ Injection<br>□ Topical product<br>□ ( )	□	Start date: Year Month Day<br>/ □ From before administration of this drug<br>End date: Year Month Day / □ Continuation	□ Treatment of adverse events (No)<br>□ Treatment of complications (No)<br>□ Prevention
12			□ PO<br>□ Injection<br>□ Topical product<br>□ ( )	□	Start date: Year Month Day<br>/ □ From before administration of this drug<br>End date: Year Month Day / □ Continuation	□ Treatment of adverse events (No)<br>□ Treatment of complications (No)<br>□ Prevention
13			□ PO<br>□ Injection<br>□ Topical product<br>□ ( )	□	Start date: Year Month Day<br>/ □ From before administration of this drug<br>End date: Year Month Day / □ Continuation	□ Treatment of adverse events (No)<br>□ Treatment of complications (No)<br>□ Prevention
14			□ PO<br>□ Injection<br>□ Topical product<br>□ ( )	□	Start date: Year Month Day<br>/ □ From before administration of this drug<br>End date: Year Month Day / □ Continuation	□ Treatment of adverse events (No)<br>□ Treatment of complications (No)<br>□ Prevention
15			□ PO<br>□ Injection<br>□ Topical product<br>□ ( )	□	Start date: Year Month Day<br>/ □ From before administration of this drug<br>End date: Year Month Day / □ Continuation	□ Treatment of adverse events (No)<br>□ Treatment of complications (No)<br>□ Prevention
16			□ PO<br>□ Injection<br>□ Topical product<br>□ ( )	□	Start date: Year Month Day<br>/ □ From before administration of this drug<br>End date: Year Month Day / □ Continuation	□ Treatment of adverse events (No)<br>□ Treatment of complications (No)<br>□ Prevention
17			□ PO<br>□ Injection<br>□ Topical product<br>□ ( )	□	Start date: Year Month Day<br>/ □ From before administration of this drug<br>End date: Year Month Day / □ Continuation	□ Treatment of adverse events (No)<br>□ Treatment of complications (No)<br>□ Prevention

Supplement :
・If the reason for use is ""treatment of adverse events,"" please fill in the details in 【Safety Evaluation】- Adverse Events (pages 9 - 11).
・If it is ""treatment of complications,"" please confirm that there is a corresponding entry in 【Patient Background】 (page 2) under ""Medical History.""
・Prevention includes, for example, gastrointestinal drugs prescribed for the prevention of gastritis.

(continued)

No.	Name of drug	Daily dose (units)	Route of administration	As needed	Treatment period (From start of administration to end or change)	Reasons for use ※Including associated symptoms
18			□ PO<br>□ Injection<br>□ Topical product<br>□ ( )	□	Start date: Year Month Day<br>/ □ From before administration of this drug<br>End date: Year Month Day / □ Continuation	□ Treatment of adverse events (No)<br>□ Treatment of complications (No)<br>□ Prevention
19			□ PO<br>□ Injection<br>□ Topical product<br>□ ( )	□	Start date: Year Month Day<br>/ □ From before administration of this drug<br>End date: Year Month Day / □ Continuation	□ Treatment of adverse events (No)<br>□ Treatment of complications (No)<br>□ Prevention
20			□ PO<br>□ Injection<br>□ Topical product<br>□ ( )	□	Start date: Year Month Day<br>/ □ From before administration of this drug<br>End date: Year Month Day / □ Continuation	□ Treatment of adverse events (No)<br>□ Treatment of complications (No)<br>□ Prevention

Supplement :
・If the reason for use is ""treatment of adverse events,"" please fill in the details in 【Safety Evaluation】- Adverse Events (pages 9 - 11).
・If it is ""treatment of complications,"" please confirm that there is a corresponding entry in 【Patient Background】 (page 2) under ""Medical History.""
・Prevention includes, for example, gastrointestinal drugs prescribed for the prevention of gastritis.

≪Other than drug therapy≫

□ Yes	□ No

No.	Treatment	Testing period (From start to end of treatment)	Reason for performance ※Including associated symptoms
1		Start date: Year Month Day<br>/ □ From before administration of this drug<br>End date: Year Month Day / □ Continuation	□ Treatment of adverse events (No)<br>□ Treatment of complications (No)<br>□ Prevention
2		Start date: Year Month Day<br>/ □ From before administration of this drug<br>End date: Year Month Day / □ Continuation	□ Treatment of adverse events (No)<br>□ Treatment of complications (No)<br>□ Prevention
3		Start date: Year Month Day<br>/ □ From before administration of this drug<br>End date: Year Month Day / □ Continuation	□ Treatment of adverse events (No)<br>□ Treatment of complications (No)<br>□ Prevention

Supplement :
・If the reason for use is ""treatment of adverse events,"" please fill in the details in 【Safety Evaluation】- Adverse Events (pages 9 - 11).
・If it is ""treatment of complications,"" please confirm that there is a corresponding entry in 【Patient Background】 (page 2) under ""Medical History.""

【 clinical course 】 -Observation for nocturia

・Please fill in the ""daily urine volume,"" ""nighttime urine volume,"" ""water consumption per day,"" ""dinner time and medication times"" for each observation period.
・The following information is important to prevent the occurrence and exacerbation of hyponatremia and is used for cause analysis, so please measure it.

Week / Scale	Before administration of this drug (4 weeks to 0 weeks before administration)	Week 12 (Week 10 to Week 14)	At discontinuation/end
	Year Month Day<br>□ Not performed	Year Month Day<br>□ Not performed	Year Month Day<br>□ Not performed
Daily urine volume	mL<br>□ Not measured	mL<br>□ Not measured	mL<br>□ Not measured
Nighttime urine volume	mL<br>□ Not measured	mL<br>□ Not measured	mL<br>□ Not measured
Water consumption per day (mL)	mL<br>□ Not measured	mL<br>□ Not measured	mL<br>□ Not measured
Dinner time and Medication times (24Hr)	Dinner time: Hour Minute<br>Medication time: Hour Minute<br>□ Unknown	Dinner time: Hour Minute<br>Medication time: Hour Minute<br>□ Unknown	Dinner time: Hour Minute<br>Medication time: Hour Minute<br>□ Unknown

【 Safety Specification 】 -Occurrence status of symptoms caused by or recognized as hyponatremia

・Please record the presence or absence of symptoms related to hyponatremia (malaise, headache, nausea/vomiting, confusion, edema, seizures, stupor/coma) during the observation period in this survey.
・Please record the presence or absence of acute diseases that may cause hyponatremia (systemic infection, fever, gastroenteritis, diarrhea) during the observation period in this survey.
・Please record the presence or absence of the above diseases and symptoms regardless of the occurrence of hyponatremia.
・If ""Yes,"" be sure to fill in the details of the adverse event in 【Safety Evaluation】- Adverse Events (pages 9-11). Also, please record the clinical test results that served as the basis for diagnosis in 【Tests】- Clinical tests related to events that should be investigated (page 5) or 【Tests】- Related to adverse events (page 12).

Name of event/symptom	presence or absence	
""Hyponatremia""<br>Presence or absence of adverse event	□ Yes(No)<br>→ Please record the clinical test results that served as the basis for diagnosis, such as ""serum sodium level,"" in 【Tests】- Clinical tests related to events that should be investigated (page 5).	□ No
""Systemic infection""<br>Presence or absence of adverse event	□ Yes(No)	□ No
""Fever""<br>Presence or absence of adverse event	□ Yes(No)<br>→ Please record the clinical test results that served as the basis for diagnosis, such as ""body temperature,"" in 【Tests】- Related to adverse events (page 12).	□ No
""Gastroenteritis""<br>Presence or absence of adverse event	□ Yes(No)	□ No
""Diarrhea""<br>Presence or absence of adverse event	□ Yes(No)	□ No
""Malaise""<br>Presence or absence of adverse event	□ Yes(No)	□ No
""Headache""<br>Presence or absence of adverse event	□ Yes(No)	□ No
""Nausea/vomiting""<br>Presence or absence of adverse event	□ Yes(No)	□ No
""Confusion""<br>Presence or absence of adverse event	□ Yes(No)	□ No
""Edema""<br>Presence or absence of adverse event	□ Yes(No)	□ No
""Seizures""<br>Presence or absence of adverse event	□ Yes(No)	□ No
""Stupor/coma""<br>Presence or absence of adverse event	□ Yes(No)	□ No

【 Safety assessment 】 - Adverse events

- Please record each event of any unfavorable or unintended sign (including abnormal laboratory findings), symptom, or disease that occurred or worsened from the start of administration of this drug to the end of the observation period.
- Please also record the progression/worsening of the underlying disease/complications (including associated symptoms) and death.
- For adverse events judged to have ""no"" causal relationship with this drug, please record the reason for denial in the comment section on page 11.

Severe Serious Degree Group Criteria	No.1. Died	Results in death
	N o2. Life-threatening	Life threatening IH
	No.3. hitch	Results in persistent or significant disability/incapacity
	No.4. Hospitalization/Prolongation of hospitalization	Requires inpatient hospitalization or prolongation of existing hospitalization.
	No.5. Serious as per No.1-4	Other medically important condition
	No.6. birth defects	Is a congenital anomaly/birth defect

If there is an event selected as No.1, please also record the cause of death in ""In case of death"" on page 11.

Criteria for determining the relationship with this drug	unrelated	If an explanation can be provided for concomitant drugs or complications other than the medicinal product in question, or if a causal relationship can be ruled out based on temporal correlation.
	yes	If the conditions for ""unrelated"" do not apply, and there are no factors to deny a causal relationship with this drug.

□ Yes □ No

No.	Name of event	In the case of a serious event, please confirm the applicable reason from the ""Severity Criteria"" and select the ""Applicable No.""			
1		Seriousness	□ Non-serious<br>Serious ↓<br>□ No.1: Died<br>□ No.2: Life-threatening<br>□ No.3: hitch<br>□ No.4: Hospitalization/Prolongation of hospitalization<br>□ No.5: Serious as per No.1-4<br>□ No.6: birth defects	Out co me	(Outcome confirmation date: Year Month Day)<br>□ Recovered □ Improved □ Not recovered<br>□ Resolved with sequelae □ Death Note) □ Unknown<br>Note) Please record the outcome as death only if this event was the cause of death. Also, please record the cause of death in ""In case of death"" at the bottom of page 11. Please select ""Not recovered"" for all events that were unresolved at the time of death.
		treatment drug · Therapy	□ No □ Yes □ Unknown<br>※If ""Yes,"" please record the details in 【Concomitant therapy for diseases other than the underlying disease during the observation period】 (pages 5-7).	Other than this drug Suspected drug (Supplement)	□ No □ Yes↓ □ Unknown<br>Select from the dropdown
		To this drug Regarding Treatment	□ Discontinuation □ Drug holiday<br>□ Dose reduction □ Dose increase<br>□ No dose modification<br>□ Treatment at another medical institution<br>※ In case of discontinuation, provide details in 【Status of drug administration at the end of observation period】 -Patient outcome (page 3)	Factors other than the drug (Supplement)	□ No □ Yes↓ □ Unknown
Onset Date : years month day)		Relation to this drug	□ Yes □ No		
2		Seriousness	□ Non-serious<br>Serious ↓<br>□ No.1: Died<br>□ No.2: Risk of death<br>□ No.3: hitch<br>□ No.4: Hospitalization/Prolonged hospitalization<br>□ No.5: Serious as per No.1-4<br>□ No.6: birth defects	Out co me	(Outcome confirmation date: Year Month Day)<br>□ Recovered □ Improved □ Not recovered<br>□ Resolved with sequelae □ Death Note) □ Unknown<br>Note) Only mark outcome as death if this event was the cause of death. Also, record the cause of death at the bottom of page 11 under 'In case of death'. Select 'Not recovered' for all unresolved events at the time of death.
		treatment drug · Therapy	□ No □ Yes □ Unknown<br>※ If yes, provide details in 【Concomitant therapy for diseases other than the primary disease during the observation period】 (pages 5-7).	Other than this drug Suspected drug (Supplement)	□ No □ Yes↓ □ Unknown<br>Select from the dropdown
		To this drug Regarding Treatment	□ Discontinuation □ Drug holiday<br>□ Dose reduction □ Dose increase<br>□ No dose modification<br>□ Treatment at another medical institution<br>※ In case of discontinuation, provide details in 【Status of drug administration at the end of observation period】 -Patient outcome (page 3)	Factors other than the drug (Supplement)	□ No □ Yes↓ □ Unknown
Onset Date : years month day)		Relation to this drug	□ Yes □ No		

Supplementary description

- If other suspected drugs are 'Yes', record the drug name and provide details in 【Concomitant therapy】 (pages 5-7). If 'No' is selected for relation to this drug, record the reason in 'Other suspected drugs' or 'Factors other than the drug'.
- In the 'Treatment regarding this drug' section, 'Discontinuation' refers to cases where treatment with this drug at the medical institution is unavoidably abandoned due to progression/aggravation of the primary disease, occurrence of adverse events, patient request, death, or transfer. 'Drug holiday' refers to cases where administration of this drug was interrupted after the previous dose but resumed during the observation period. If administration is resumed with a reduced dose after interruption, select 'Dose reduction'.

【 Safety assessment 】 - Adverse events (continued)

No.	Name of event	For serious events, confirm the applicable reason from the 'Seriousness criteria' and select the 'Applicable No.'.			
3		Seriousness	□ Non-serious<br>Serious ↓<br>□ No.1: Died<br>□ No.2: Risk of death<br>□ No.3: hitch<br>□ No.4: Hospitalization/Prolonged hospitalization<br>□ No.5: Serious as per No.1-4<br>□ No.6: birth defects	Outcome	(Outcome confirmation date: Year Month Day)<br>□ Recovered □ Improved □ Not recovered<br>□ Resolved with sequelae □ Death Note) □ Unknown
					Note) Only mark outcome as death if this event was the cause of death. Also, record the cause of death at the bottom of page 11 under 'In case of death'. Select 'Not recovered' for all unresolved events at the time of death.
		treatment drug ・Therapy	□ No □ Yes □ Unknown<br>※ If yes, provide details in 【Concomitant therapy for diseases other than the primary disease during the observation period】 (pages 5-7).	Other than this drug Suspected drug (Supplement)	□ No □ Yes↓ □ Unknown<br>Select from the dropdown
		To this drug Regarding Treatment	□ Discontinuation □ Drug holiday<br>□ Dose reduction □ Dose increase<br>□ No dose modification<br>□ Treatment at another medical institution<br>※ In case of discontinuation, provide details in 【Status of drug administration at the end of observation period】 -Patient outcome (page 3)	Factors other than the drug (Supplement)	□ No □ Yes↓ □ Unknown
Onset Date :<br>years month day)		Relation to this drug	□ Yes □ No		
4		Seriousness	□ Non-serious<br>Serious ↓<br>□ No.1: Died<br>□ No.2: Risk of death<br>□ No.3: hitch<br>□ No.4: Hospitalization/Prolonged hospitalization<br>□ No.5: Serious as per No.1-4<br>□ No.6: birth defects	Outcome	(Outcome confirmation date: Year Month Day)<br>□ Recovered □ Improved □ Not recovered<br>□ Resolved with sequelae □ Death Note) □ Unknown
					Note) Only mark outcome as death if this event was the cause of death. Also, record the cause of death at the bottom of page 11 under 'In case of death'. Select 'Not recovered' for all unresolved events at the time of death.
		treatment drug	□ No □ Yes □ Unknown<br>※ If yes, provide details in 【Concomitant therapy for diseases other than the primary disease during the observation period】 (pages 5-7).	Other than this drug Suspected drug (Supplement)	□ No □ Yes↓ □ Unknown<br>Select from the dropdown
		To this drug Regarding Treatment	□ Discontinuation □ Drug holiday<br>□ Dose reduction □ Dose increase<br>□ No dose modification<br>□ Treatment at another medical institution<br>※ In case of discontinuation, provide details in 【Status of drug administration at the end of observation period】 -Patient outcome (page 3)	Factors other than the drug (Supplement)	□ No □ Yes↓ □ Unknown
Onset Date :<br>years month day)		Relation to this drug	□ Yes □ No		
5		Seriousness	□ Non-serious<br>Serious ↓<br>□ No.1: Died<br>□ No.2: Risk of death<br>□ No.3: hitch<br>□ No.4: Hospitalization/Prolonged hospitalization<br>□ No.5: Serious as per No.1-4<br>□ No.6: birth defects	Outcome	(Outcome confirmation date: Year Month Day)<br>□ Recovered □ Improved □ Not recovered<br>□ Resolved with sequelae □ Death Note) □ Unknown
					Note) Only mark outcome as death if this event was the cause of death. Also, record the cause of death at the bottom of page 11 under 'In case of death'. Select 'Not recovered' for all unresolved events at the time of death.
		treatment drug ・Therapy	□ No □ Yes □ Unknown<br>※ If yes, provide details in 【Concomitant therapy for diseases other than the primary disease during the observation period】 (pages 5-7).	Other than this drug Suspected drug (Supplement)	□ No □ Yes↓ □ Unknown<br>Select from the dropdown
		To this drug Regarding Treatment	□ Discontinuation □ Drug holiday<br>□ Dose reduction □ Dose increase<br>□ No dose modification<br>□ Treatment at another medical institution<br>※ In case of discontinuation, provide details in 【Status of drug administration at the end of observation period】 -Patient outcome (page 3)	Factors other than the drug (Supplement)	□ No □ Yes↓ □ Unknown
Onset Date :<br>years month day)		Relation to this drug	□ Yes □ No		

Supplementary description: If other suspected drugs are 'Yes', record the drug name and provide details in 【Concomitant therapy】 (pages 5-7). If 'No' is selected for relation to this drug, record the reason in 'Other suspected drugs' or 'Factors other than the drug'.

【 Safety assessment 】 - Adverse events (continued)

No.	Name of event	For serious events, confirm the applicable reason from the 'Seriousness criteria' and select the 'Applicable No.'.			
6		Seriousness	□ Non-serious<br>Serious ↓<br>□ No.1: Died<br>□ No.2: Risk of death<br>□ No.3: hitch<br>□ No.4: Hospitalization/Prolonged hospitalization<br>□ No.5: Serious as per No.1-4<br>□ No.6: birth defects	Outcome	(Outcome confirmation date: Year Month Day)<br>□ Recovered □ Improved □ Not recovered<br>□ Resolved with sequelae □ Death Note) □ Unknown
					Note) Only mark outcome as death if this event was the cause of death. Also, record the cause of death at the bottom of page 11 under 'In case of death'. Select 'Not recovered' for all unresolved events at the time of death.
		treatment drug ・Therapy	□ No □ Yes □ Unknown<br>※ If yes, provide details in 【Concomitant therapy for diseases other than the primary disease during the observation period】 (pages 5-7).	Other than this drug Suspected drug (Supplement)	□ No □ Yes↓ □ Unknown<br>Select from the dropdown
		To this drug Regarding Treatment	□ Discontinuation □ Drug holiday<br>□ Dose reduction □ Dose increase<br>□ No dose modification<br>□ Treatment at another medical institution<br>※ In case of discontinuation, provide details in 【Status of drug administration at the end of observation period】 -Patient outcome (page 3)	Factors other than the drug (Supplement)	□ No □ Yes↓ □ Unknown
Onset Date : years month day)		Relation to this drug	□ Yes □ No		
7		Seriousness	□ Non-serious<br>Serious ↓<br>□ No.1: Died<br>□ No.2: Risk of death<br>□ No.3: hitch<br>□ No.4: Hospitalization/Prolonged hospitalization<br>□ No.5: Serious as per No.1-4<br>□ No.6: birth defects	Outcome	(Outcome confirmation date: Year Month Day)<br>□ Recovered □ Improved □ Not recovered<br>□ Resolved with sequelae □ Death Note) □ Unknown
					Note) Only mark outcome as death if this event was the cause of death. Also, record the cause of death at the bottom of page 11 under 'In case of death'. Select 'Not recovered' for all unresolved events at the time of death.
		treatment drug ・Therapy	□ No □ Yes □ Unknown<br>※ If yes, provide details in 【Concomitant therapy for diseases other than the primary disease during the observation period】 (pages 5-7).	Other than this drug Suspected drug (Supplement)	□ No □ Yes↓ □ Unknown<br>Select from the dropdown
		To this drug Regarding Treatment	□ Discontinuation □ Drug holiday<br>□ Dose reduction □ Dose increase<br>□ No dose modification<br>□ Treatment at another medical institution<br>※ In case of discontinuation, provide details in 【Status of drug administration at the end of observation period】 -Patient outcome (page 3)	Factors other than the drug (Supplement)	□ No □ Yes↓ □ Unknown
Onset Date : years month day)		Relation to this drug	□ Yes □ No		

Supplementary description: If other suspected drugs are 'Yes', record the drug name and provide details in 【Concomitant therapy】 (pages 5-7). If 'No' is selected for relation to this drug, record the reason in 'Other suspected drugs' or 'Factors other than the drug'.

In case of death **Record the cause of death and autopsy details. Ensure the cause of death matches the event name recorded for the applicable adverse event.**

Date of death	Year Month Day	Investigations post mortem	to perform ⇒<br>□ Not performed<br>□ Unknown	autopsy finding :
cause of death	Select from the dropdown			

For adverse events judged as 'No' causal relationship with this drug above, record the reason for denial in the comments field.

comments field	

If 'Treatment at another medical institution' is selected for adverse event management above, record the following information.

Which medical institution is providing treatment?	□ Hospital name ( ) □ Unknown
Is it permissible to confirm details with the physician at that medical institution?	□ Permitted □ Not Permitted

【 test 】 - Related to adverse events

・ Please describe the results of clinical tests related to adverse events. At that time, please also include the clinical test results from before administration of this drug to the confirmation of the outcome (including the worst test values).

No.	Timepoint / Test item and unit		Baseline	After administration Course from the occurrence of the relevant event to the confirmation of the outcome (including the worst test values)				
			years month day	years month day	years month day	years month day	years month day	years month day
1								
2								
3								
4								
5								
6								
7								
8								
9								
10								
11								
12								
13								
14								
15								
16								
17								
18								
19								
20								

【 hyponatraemia 】 - Investigation items at the time of hyponatremia occurrence

・If the adverse event of ""hyponatremia"" occurs, please describe the following situations at the time of occurrence.
・This information is necessary for the assessment of hyponatremia and will also be used for cause analysis, so please obtain and record the following information from the patient at the time of occurrence: presence of symptoms (fatigue, headache, nausea/vomiting, confusion, edema, seizures, stupor/coma, etc.), presence of acute diseases at the time of occurrence (systemic infections, fever, gastroenteritis, etc.).

Subgroup	which way the wind blows
Fluid intake status before and after administration of this drug (drinking water, drip/infusion)	Please describe the specific situation ( )
Daily urine volume before and after the occurrence of hyponatremia	20 years month day mL □ Not measured<br>20 years month day mL □ Not measured
presence of symptoms	□ No<br>Yes ⇒ Please describe the specific symptoms ( )
Presence of acute diseases at the time of occurrence	□ No<br>Yes ⇒ Please describe the specific symptoms ( )
Dinner time immediately before occurrence and medication time of this drug (24Hr)	Dinner time: hour minute<br>Medication time of this drug: hour minute
