# Supplementary material for: Long‐Term Safety of Desmopressin Orally Disintegrating Tablets in Men With Nocturia due to Nocturnal Polyuria: Final Results of a Specified Drug Use‐Results Survey in Japan
Source: Low Urin Tract Symptoms. 2026 Mar 17;18(2):e70052. doi: 10.1111/luts.70052 (PMC12995508; doi:10.1111/luts.70052)
Supplement: Supplementary file 2 — File S2: CRF2_Data for 13–52 weeks after the start of administration of this drug. [file LUTS-18-e70052-s001.pdf]

≪CRF 2 "Data for 13-52 weeks after the start of administration of this drug" (Total 12 pages)≫

【 patient characteristics 】 -Patient Identification

・Please enter the patient demographics at the start of administration of this drug.

Patient's Initials ※1	Last name	First name	Sex	■ Male	Date of Birth or Age ※2 (at the start of administration of this drug)	□ Taisho □ Showa □ Heisei □ Gregorian Calendar Year Month Day	patient to identify No.	()
						years		

※If you cannot provide the patient's initials, please enter XX.
※From a privacy protection standpoint, if you cannot provide the complete date of birth, please enter up to the year and month.

【 records on dosing 】 - Use of this Drug

- Please enter the usage status after the start of administration of this drug (during the observation period in this survey form).
- If the regimen is changed, please enter it separately in another row. If treatment is continued with the same regimen, do not enter the "end date" and check "Ongoing."

No.	Daily dose	Treatment Period (from start of administration to end of administration or date of regimen change)			Reason for Dose Modification
1	□ 25μg<br>□ 50μg<br>□ ()μg	Year Month Day<br>/□ Continued from previous volume	～	20 Year Month Day<br>/ □ Continued	AE(No)<br>□ Reasons other than adverse events
2	□ 25μg<br>□ 50μg<br>□ ()μg	Year Month Day	～	Year Month Day<br>/ □ Continued	AE(No)<br>□ Reasons other than adverse events
3	□ 25μg<br>□ 50μg<br>□ ()μg	Year Month Day	～	Year Month Day<br>/ □ Continued	AE(No)<br>□ Reasons other than adverse events
4	□ 25μg<br>□ 50μg<br>□ ()μg	Year Month Day	～	Year Month Day<br>/ □ Continued	AE(No)<br>□ Reasons other than adverse events
5	□ 25μg<br>□ 50μg<br>□ ()μg	Year Month Day	～	Year Month Day<br>/ □ Continued	AE(No)<br>□ Reasons other than adverse events
6	□ 25μg<br>□ 50μg<br>□ ()μg	Year Month Day	～	Year Month Day<br>/ □ Continued	AE(No)<br>□ Reasons other than adverse events
7	□ 25μg<br>□ 50μg<br>□ ()μg	Year Month Day	～	Year Month Day<br>/ □ Continued	AE(No)<br>□ Reasons other than adverse events
8	□ 25μg<br>□ 50μg<br>□ ()μg	Year Month Day	～	Year Month Day<br>/ □ Continued	AE(No)<br>□ Reasons other than adverse events
9	□ 25μg<br>□ 50μg<br>□ ()μg	Year Month Day	～	Year Month Day<br>/ □ Continued	AE(No)<br>□ Reasons other than adverse events
10	□ 25μg<br>□ 50μg<br>□ ()μg	Year Month Day	～	Year Month Day<br>/ □ Continued	AE(No)<br>□ Reasons other than adverse events
11	□ 25μg<br>□ 50μg<br>□ ()μg	Year Month Day	～	Year Month Day<br>/ □ Continued	AE(No)<br>□ Reasons other than adverse events
12	□ 25μg<br>□ 50μg<br>□ ()μg	Year Month Day	～	Year Month Day<br>/ □ Continued	

Note: If the reason for regimen change or drug holiday is "Adverse Event," please enter details in 【Safety Evaluation】 (pages 8 - 10).

【Administration Status of this Drug at the End of the Observation Period】 - Patient Outcomes

・Please enter the medical classification, administration status, and progress information at the end of the observation period in this survey form.

date confirmed	(Year Month Day)	
Medical Classification	□ Hospitalization ※ □ Outpatient □ Unknown<br>※If the reason for hospitalization is "Occurrence and treatment of adverse events," please be sure to enter details in 【Safety Evaluation】 (pages 8 - 10).	
Use of secukinumab	□ Ongoing	
	□ Ended (End Date: Year Month Day)	
	□ Discontinued (Discontinuation Date: Year Month Day)	
	〔Reason for Discontinuation〕	□ Patient's request (for reasons other than adverse events)
	※Select one	□ Adverse Event ⇒ Please enter details in 【Safety Evaluation】 (pages 8 - 10). 〔Adverse Event No.: 〕<br>Progression/aggravation of primary disease/complications (including accompanying symptoms), death<br>【 Safety assessment 】 - Please record in the Adverse Event(s) page.
		□ Lack of efficacy
		□ Transfer (Transfer Date: Year Month Day)
		□ Did not attend (Last Visit Date: Year Month Day)
		□ Other (Details:)

Note: "Ended" refers to cases where treatment with this drug was terminated due to improvement (marked efficacy, etc.), while "Discontinued" refers to cases where treatment with this drug was unavoidably abandoned at the medical institution due to progression/aggravation of the primary disease, occurrence of adverse events, patient's request, death, transfer, etc.

【 Combination Therapy 】 - Treatment drugs for nocturia other than this drug

・Please enter information on treatment drugs for nocturia other than this drug (pharmacotherapy) during the observation period in this survey form. If the regimen is changed, please enter it separately in another row. In the case of as-needed medication, please enter the single dose in the Daily Dose column.
・If treatment is continued with the same regimen, do not enter the "end date" and check "Ongoing."

□ Yes	□ No

No.	Name of drug	Daily dose (units)	Route of administration	As-needed	Treatment period (from start of administration to end of administration or change)
1			□ PO<br>□ Injection<br>□ Topical<br>□ ( )	□	Start Date: Year Month Day / □ Continued from previous volume<br>End Date: Year Month Day / □ Ongoing
2			□ PO<br>□ Injection<br>□ Topical<br>□ ( )	□	Start Date: Year Month Day / □ Continued from previous volume<br>End Date: Year Month Day / □ Ongoing
3			□ PO<br>□ Injection<br>□ Topical<br>□ ( )	□	Start Date: Year Month Day / □ Continued from previous volume<br>End Date: Year Month Day / □ Ongoing
4			□ PO<br>□ Injection<br>□ Topical<br>□ ( )	□	Start Date: Year Month Day / □ Continued from previous volume<br>End Date: Year Month Day / □ Ongoing
5			□ PO<br>□ Injection<br>□ Topical<br>□ ( )	□	Start Date: Year Month Day / □ Continued from previous volume<br>End Date: Year Month Day / □ Ongoing

【Combination Therapy】 - Combination Therapy for Nocturia

- Please enter information on combination therapy for nocturia other than pharmacotherapy during the observation period in this survey form.
- For drug treatment for nocturia, please enter it under 【Combination Therapy】- treatment drugs for nocturia other than this drug (page 2).
- If the same treatment is to be continued at the end of the observation period of this survey form, do not fill in the 'end date' and check 'continuation'.

□ Yes	□ No

No.	Treatment	Testing period (from the start of treatment to the end of treatment)
1	□ Behavioral therapy<br>□ exercise therapy<br>Other ( )	Start date: Year Month Day / □ Continued from previous volume<br>End date: Year Month Day / □ Continuation
2	□ Behavioral therapy<br>□ exercise therapy<br>Other ( )	Start date: Year Month Day / □ Continued from previous volume<br>End date: Year Month Day / □ Continuation
3	□ Behavioral therapy<br>□ exercise therapy<br>Other ( )	Start date: Year Month Day / □ Continued from previous volume<br>End date: Year Month Day / □ Continuation
4	□ Behavioral therapy<br>□ exercise therapy<br>Other ( )	Start date: Year Month Day / □ Continued from previous volume<br>End date: Year Month Day / □ Continuation
5	□ Behavioral therapy<br>□ exercise therapy<br>Other ( )	Start date: Year Month Day / □ Continued from previous volume<br>End date: Year Month Day / □ Continuation

【test】 -Clinical tests related to events that should be investigated intensively

- If the following test items were conducted during administration, please enter the clinical test results.
- Regardless of the causal relationship with drug administration, if judged to be clinically undesirable abnormal fluctuations, please enter it in 【Safety Evaluation】-Adverse Events (pages 8-10).

No.	Timepoint / Test item	Unit	during the drug administration		
			Week 24 (Week 20 to Week 28)	Week 52 (Week 48 to Week 56)	at discontinuation/termination
			Year Month Day<br>□ Not tested	Year Month Day<br>□ Not tested	Year Month Day<br>□ Not tested
1	serum creatinine	mg/dL			
2	White blood cell count	/μL			
3	monocyte count/segment	□ /μL<br>□ %			
4	serum sodium	mmol/L			
5	Urea nitrogen (BUN)	mg/dL			
6	Blood BNP	pg/mL			
7	NT-proBNP	pg/mL			

【 Combination Therapy 】 -Combination therapy for diseases other than the primary disease during the observation period

・Please enter the combination therapy (drug therapy, non-drug therapy) during the observation period in this survey form.
・For drug therapy, if the dosage and administration are changed, please enter it separately in another row. In the case of as-needed medication, please enter the single dose in the daily dose column.
・If the same treatment (in the case of drug treatment, with the same dosage and administration) is to be continued, do not fill in the 'end date' and check 'continuation'.
・Please also enter the adverse event treatment drugs and adverse event treatment therapies.
・Please enter all information regarding drips and infusions.
・For drug treatment for nocturia, please enter it under 【Combination Therapy】- treatment drugs for nocturia other than this drug (page 2).
・For combination therapy for nocturia other than drug therapy, please enter it under 【Combination Therapy】-Combination therapy for nocturia (page 3).
・Since it is listed as a risk factor for hyponatremia, be sure to enter it if bone disease treatment drugs and hyperlipidemia treatment drugs are being administered.

≪ Drug therapy ≫

□ Yes	□ No

No.	Name of drug	Daily dose (units)	Route of administration	As needed	Treatment period (from the start of administration to the end or change of administration)	Reasons for use ※including associated symptoms
1			□ PO<br>□ Injection<br>□ Topical product<br>□ ( )	□	Start date: Year Month Day<br>/ □ Continued from previous volume<br>End date: Year Month Day / □ Continuation	□ Treatment of adverse events (No)<br>□ Treatment of complications (No)<br>□ Prevention
2			□ PO<br>□ Injection<br>□ Topical product<br>□ ( )	□	Start date: Year Month Day<br>/ □ Continued from previous volume<br>End date: Year Month Day / □ Continuation	□ Treatment of adverse events (No)<br>□ Treatment of complications (No)<br>□ Prevention
3			□ PO<br>□ Injection<br>□ Topical product<br>□ ( )	□	Start date: Year Month Day<br>/ □ Continued from previous volume<br>End date: Year Month Day / □ Continuation	□ Treatment of adverse events (No)<br>□ Treatment of complications (No)<br>□ Prevention
4			□ PO<br>□ Injection<br>□ Topical product<br>□ ( )	□	Start date: Year Month Day<br>/ □ Continued from previous volume<br>End date: Year Month Day / □ Continuation	□ Treatment of adverse events (No)<br>□ Treatment of complications (No)<br>□ Prevention
5			□ PO<br>□ Injection<br>□ Topical product<br>□ ( )	□	Start date: Year Month Day<br>/ □ Continued from previous volume<br>End date: Year Month Day / □ Continuation	□ Treatment of adverse events (No)<br>□ Treatment of complications (No)<br>□ Prevention
6			□ PO<br>□ Injection<br>□ Topical product<br>□ ( )	□	Start date: Year Month Day<br>/ □ Continued from previous volume<br>End date: Year Month Day / □ Continuation	□ Treatment of adverse events (No)<br>□ Treatment of complications (No)<br>□ Prevention
7			□ PO<br>□ Injection<br>□ Topical product<br>□ ( )	□	Start date: Year Month Day<br>/ □ Continued from previous volume<br>End date: Year Month Day / □ Continuation	□ Treatment of adverse events (No)<br>□ Treatment of complications (No)<br>□ Prevention
8			□ PO<br>□ Injection<br>□ Topical product<br>□ ( )	□	Start date: Year Month Day<br>/ □ Continued from previous volume<br>End date: Year Month Day / □ Continuation	□ Treatment of adverse events (No)<br>□ Treatment of complications (No)<br>□ Prevention
9			□ PO<br>□ Injection<br>□ Topical product<br>□ ( )	□	Start date: Year Month Day<br>/ □ Continued from previous volume<br>End date: Year Month Day / □ Continuation	□ Treatment of adverse events (No)<br>□ Treatment of complications (No)<br>□ Prevention
10			□ PO<br>□ Injection<br>□ Topical product<br>□ ( )	□	Start date: Year Month Day<br>/ □ Continued from previous volume<br>End date: Year Month Day / □ Continuation	□ Treatment of adverse events (No)<br>□ Treatment of complications (No)<br>□ Prevention
11			□ PO<br>□ Injection<br>□ Topical product<br>□ ( )	□	Start date: Year Month Day<br>/ □ Continued from previous volume<br>End date: Year Month Day / □ Continuation	□ Treatment of adverse events (No)<br>□ Treatment of complications (No)<br>□ Prevention

Supplement:
・If the reason for use is 'treatment of adverse events', please enter details in 【Safety Evaluation】-Adverse Events (pages 8-10).
・If it is 'treatment of complications', please confirm that there is a corresponding entry in the 'medical history' of the 12-week survey form 【Patient Background】 (page 2).
・Prevention includes, for example, gastrointestinal drugs prescribed for the prevention of gastritis.

(continued)

No.	Name of drug	Daily dose (units)	Route of administration	As needed	Treatment period (from the start of administration to the end or change of administration)	Reasons for use ※including associated symptoms
12			□ PO<br>□ Injection<br>□ Topical product<br>□ ( )	□	Start date: Year Month Day<br>/ □ Continued from previous volume<br>End date: Year Month Day / □ Continuation	□ Treatment of adverse events (No)<br>□ Treatment of complications (No)<br>□ Prevention
13			□ PO<br>□ Injection<br>□ Topical product<br>□ ( )	□	Start date: Year Month Day<br>/ □ Continued from previous volume<br>End date: Year Month Day / □ Continuation	□ Treatment of adverse events (No)<br>□ Treatment of complications (No)<br>□ Prevention
14			□ PO<br>□ Injection<br>□ Topical product<br>□ ( )	□	Start date: Year Month Day<br>/ □ Continued from previous volume<br>End date: Year Month Day / □ Continuation	□ Treatment of adverse events (No)<br>□ Treatment of complications (No)<br>□ Prevention
15			□ PO<br>□ Injection<br>□ Topical product<br>□ ( )	□	Start date: Year Month Day<br>/ □ Continued from previous volume<br>End date: Year Month Day / □ Continuation	□ Treatment of adverse events (No)<br>□ Treatment of complications (No)<br>□ Prevention
16			□ PO<br>□ Injection<br>□ Topical product<br>□ ( )	□	Start date: Year Month Day<br>/ □ Continued from previous volume<br>End date: Year Month Day / □ Continuation	□ Treatment of adverse events (No)<br>□ Treatment of complications (No)<br>□ Prevention
17			□ PO<br>□ Injection<br>□ Topical product<br>□ ( )	□	Start date: Year Month Day<br>/ □ Continued from previous volume<br>End date: Year Month Day / □ Continuation	□ Treatment of adverse events (No)<br>□ Treatment of complications (No)<br>□ Prevention
18			□ PO<br>□ Injection<br>□ Topical product<br>□ ( )	□	Start date: Year Month Day<br>/ □ Continued from previous volume<br>End date: Year Month Day / □ Continuation	□ Treatment of adverse events (No)<br>□ Treatment of complications (No)<br>□ Prevention
19			□ PO<br>□ Injection<br>□ Topical product<br>□ ( )	□	Start date: Year Month Day<br>/ □ Continued from previous volume<br>End date: Year Month Day / □ Continuation	□ Treatment of adverse events (No)<br>□ Treatment of complications (No)<br>□ Prevention
20			□ PO<br>□ Injection<br>□ Topical product<br>□ ( )	□	Start date: Year Month Day<br>/ □ Continued from previous volume<br>End date: Year Month Day / □ Continuation	□ Treatment of adverse events (No)<br>□ Treatment of complications (No)<br>□ Prevention
21			□ PO<br>□ Injection<br>□ Topical product<br>□ ( )	□	Start date: Year Month Day<br>/ □ Continued from previous volume<br>End date: Year Month Day / □ Continuation	□ Treatment of adverse events (No)<br>□ Treatment of complications (No)<br>□ Prevention
22			□ PO<br>□ Injection<br>□ Topical product<br>□ ( )	□	Start date: Year Month Day<br>/ □ Continued from previous volume<br>End date: Year Month Day / □ Continuation	□ Treatment of adverse events (No)<br>□ Treatment of complications (No)<br>□ Prevention
23			□ PO<br>□ Injection<br>□ Topical product<br>□ ( )	□	Start date: Year Month Day<br>/ □ Continued from previous volume<br>End date: Year Month Day / □ Continuation	□ Treatment of adverse events (No)<br>□ Treatment of complications (No)<br>□ Prevention
24			□ PO<br>□ Injection<br>□ Topical product<br>□ ( )	□	Start date: Year Month Day<br>/ □ Continued from previous volume<br>End date: Year Month Day / □ Continuation	□ Treatment of adverse events (No)<br>□ Treatment of complications (No)<br>□ Prevention
25			□ PO<br>□ Injection<br>□ Topical product<br>□ ( )	□	Start date: Year Month Day<br>/ □ Continued from previous volume<br>End date: Year Month Day / □ Continuation	□ Treatment of adverse events (No)<br>□ Treatment of complications (No)<br>□ Prevention
26			□ PO<br>□ Injection<br>□ Topical product<br>□ ( )	□	Start date: Year Month Day<br>/ □ Continued from previous volume<br>End date: Year Month Day / □ Continuation	□ Treatment of adverse events (No)<br>□ Treatment of complications (No)<br>□ Prevention

Supplement :
・If the reason for use is 'treatment of adverse events', please enter details in 【Safety Evaluation】-Adverse Events (pages 8-10).
・If it is 'treatment of complications', please confirm that there is a corresponding entry in the 'medical history' of the 12-week survey form 【Patient Background】 (page 2).
・Prevention includes, for example, gastrointestinal drugs prescribed for the prevention of gastritis.

(continued)

No.	Name of drug	Daily dose (units)	Route of administration	As needed	Treatment period (from the start of administration to the end or change of administration)	Reasons for use ※including associated symptoms
27			□ PO<br>□ Injection<br>□ Topical product<br>□ ( )	□	Start date: Year Month Day<br>/ □ Continued from previous volume<br>End date: Year Month Day / □ Continuation	□ Treatment of adverse events (No)<br>□ Treatment of complications (No)<br>□ Prevention
28			□ PO<br>□ Injection<br>□ Topical product<br>□ ( )	□	Start date: Year Month Day<br>/ □ Continued from previous volume<br>End date: Year Month Day / □ Continued	□ Treatment of adverse events (No)<br>□ Treatment of complications (No)<br>□ Prevention
29			□ PO<br>□ Injection<br>□ Topical product<br>□ ( )	□	Start date: Year Month Day<br>/ □ Continued from previous volume<br>End date: Year Month Day / □ Continued	□ Treatment of adverse events (No)<br>□ Treatment of complications (No)<br>□ Prevention
30			□ PO<br>□ Injection<br>□ Topical product<br>□ ( )	□	Start date: Year Month Day<br>/ □ Continued from previous volume<br>End date: Year Month Day / □ Continued	□ Treatment of adverse events (No)<br>□ Treatment of complications (No)<br>□ Prevention

Supplement :
・If the reason for use is 'treatment of adverse events', please fill in the details in 【Safety Evaluation】 - Adverse events (pages 8 - 10).
・If it is 'treatment of complications', please confirm that there is a corresponding entry in the 'medical history' of the 12-week survey form 【Patient Background】 (page 2).
・Prevention includes, for example, gastrointestinal drugs prescribed for the prevention of gastritis.

≪Other than drug therapy≫

□ Yes	□ No

No.	Treatment	Testing period (From the start of treatment to the end of treatment)	Reason for implementation ※Including associated symptoms
1		Start date: Year Month Day<br>/ □ Continued from previous volume<br>End date: Year Month Day / □ Continued	□ Treatment of adverse events (No)<br>□ Treatment of complications (No)<br>□ Prevention
2		Start date: Year Month Day<br>/ □ Continued from previous volume<br>End date: Year Month Day / □ Continued	□ Treatment of adverse events (No)<br>□ Treatment of complications (No)<br>□ Prevention
3		Start date: Year Month Day<br>/ □ Continued from previous volume<br>End date: Year Month Day / □ Continued	□ Treatment of adverse events (No)<br>□ Treatment of complications (No)<br>□ Prevention

Supplement :
・If the reason for use is 'treatment of adverse events', please fill in the details in 【Safety Evaluation】 - Adverse events (pages 8 - 10).
・If it is 'treatment of complications', please confirm that there is a corresponding entry in the 'medical history' of the 12-week survey form 【Patient Background】 (page 2).

【 clinical course 】 - Follow-up observation for nocturia

・Please fill in the 'daily urine volume', 'nighttime urine volume', 'water consumption per day', 'dinner time and medication times' for each observation period.
・The following information is important to prevent the occurrence and exacerbation of hyponatremia and is also used for cause analysis, so please measure it.

Week / Scale	Week 24 (Week 20 to Week 28)	Week 52 (Week 48 to Week 56)	At discontinuation/termination
	Year Month Day<br>□ Not performed	Year Month Day<br>□ Not performed	Year Month Day<br>□ Not performed
Daily urine volume	mL<br>□ Not measured	mL<br>□ Not measured	mL<br>□ Not measured
Nighttime urine volume	mL<br>□ Not measured	mL<br>□ Not measured	mL<br>□ Not measured
Water consumption per day (mL)	mL<br>□ Not measured	mL<br>□ Not measured	mL<br>□ Not measured
Dinner time and Medication times (24Hr)	Dinner time: Hour Minute<br>Medication time: Hour Minute<br>□ Unknown	Dinner time: Hour Minute<br>Medication time: Hour Minute<br>□ Unknown	Dinner time: Hour Minute<br>Medication time: Hour Minute<br>□ Unknown

【 Safety Specification 】 - Occurrence status of symptoms recognized as causes of hyponatremia and hyponatremia

・Please record the presence or absence of symptoms related to hyponatremia (malaise, headache, nausea/vomiting, confusion, edema, seizures, stupor/coma) during the observation period of this survey form.
・Please record the presence or absence of acute diseases that may cause hyponatremia (systemic infection, fever, gastroenteritis, diarrhea) during the observation period of this survey form.
・Please record the presence or absence of the above diseases and symptoms regardless of the occurrence of hyponatremia.
・If 'Yes', please be sure to fill in the details of the adverse event in 【Safety Evaluation】 - Adverse events (pages 8 - 10). Also, please record the clinical test results that served as the basis for diagnosis in 【Tests】 - Clinical tests related to events that should be investigated intensively (page 3) or 【Tests】 - Related to adverse events (page 11).

Name of event/symptom	presence or absence	
Of 'Hyponatremia'<br>Presence or absence of adverse event occurrence	□ Yes(No)<br>→ Please record clinical test results such as 'serum sodium level' that served as the basis for diagnosis in 【Tests】 - Clinical tests related to events that should be investigated intensively (page 3)	□ No
Of 'Systemic infection'<br>Presence or absence of adverse event occurrence	□ Yes(No)	□ No
Of 'Fever'<br>Presence or absence of adverse event occurrence	□ Yes(No)<br>→ Please record clinical test results such as 'body temperature' that served as the basis for diagnosis in 【Tests】 - Related to adverse events (page 11)	□ No
Of 'Gastroenteritis'<br>Presence or absence of adverse event occurrence	□ Yes(No)	□ No
Of 'Diarrhea'<br>Presence or absence of adverse event occurrence	□ Yes(No)	□ No
Of 'Malaise'<br>Presence or absence of adverse event occurrence	□ Yes(No)	□ No
Of 'Headache'<br>Presence or absence of adverse event occurrence	□ Yes(No)	□ No
Of 'Nausea/vomiting'<br>Presence or absence of adverse event occurrence	□ Yes(No)	□ No
Of 'Confusion'<br>Presence or absence of adverse event occurrence	□ Yes(No)	□ No
Of 'Edema'<br>Presence or absence of adverse event occurrence	□ Yes(No)	□ No
Of 'Seizures'<br>Presence or absence of adverse event occurrence	□ Yes(No)	□ No
Of 'Stupor/coma'<br>Presence or absence of adverse event occurrence	□ Yes(No)	□ No

【 Safety assessment 】 - Adverse events

- Please record each event of any unfavorable or unintended signs (including abnormal laboratory findings), symptoms, or diseases that occurred or worsened from the start of administration of this drug to the end of the observation period.
- Please also record the progression or worsening of the primary disease/complications (including associated symptoms) and death.
- For adverse events judged to have 'no' causal relationship with this drug, please record the reason for denial in the comment section on page 10.

Severe Serious Degree group Criteria	No.1. Died	Results in death	If there is an event selected as No.1, please also record the cause of death in 'In case of death' on page 10.
	N o.2. Life-threatening	Life threatening IH	
	No.3. hitch	Results in persistent or significant disability/incapacity	
	No.4. Hospitalization/Prolongation of hospitalization	Requires inpatient hospitalization or prolongation of existing hospitalization.	
	No.5. Serious as per No.1-4	Other medically important condition	
	No.6. birth defects	Is a congenital anomaly/birth defect	

Criteria for determining the relationship with this drug	unrelated	When explanations other than the drug in question, such as concomitant drugs or complications, can be provided, or when a causal relationship can be ruled out based on temporal correlation.
	yes	When the conditions for 'unrelated' do not apply, or when there are no factors to deny a causal relationship with this drug.

□ Yes	□ No

No.	Name of event	In the case of a serious event, please confirm the applicable reason from the 'Severity Criteria' and select the 'Applicable No.'.			
1		Seriousness	□ Non-serious<br>Serious ↓<br>□ No.1: Died<br>□ No.2: Life threatening<br>□ No.3: hitch<br>□ No.4: Hospitalization/prolongation of hospitalization<br>□ No.5: Serious as per No.1-4<br>□ No.6: birth defects	Outcome	(Outcome confirmation date: Year Month Day)<br>□ Resolved □ Improved □ Not resolved<br>□ Resolved with sequelae □ Death Note) □ Unknown<br>Note) Please consider the outcome as death only if this event was the cause of death. Also, please record the cause of death in 'In case of death' at the bottom of page 10. Please select 'Not resolved' for all unresolved events at the time of death.
		treatment drug・Therapy	□ No □ Yes □ Unknown<br>※ If 'Yes', please fill in the details in 【Concomitant therapy for diseases other than the primary disease during the observation period】 (pages 4-6).	Other than this drug Suspected drug (Supplement)	□ No □ Yes ↓ □ Unknown<br>Select from dropdown
		For this drug Regarding Treatment	□ Discontinuation □ Drug holiday<br>□ Dose reduction □ Dose increase<br>□ No change in administration<br>□ Treatment at another medical institution<br>※ In case of discontinuation, please fill in the details in 【Status of administration of this drug at the end of the observation period】 - Patient outcome (page 2)	Factors other than drugs (Supplement)	□ No □ Yes ↓ □ Unknown
Onset Date : Year Month Day)		Relationship with this drug	□ Yes □ No		
2		Seriousness	□ Non-serious<br>Serious ↓<br>□ No.1: Died<br>□ No.2: Life threatening<br>□ No.3: hitch<br>□ No.4: Hospitalization/prolongation of hospitalization<br>□ No.5: Serious as per No.1-4<br>□ No.6: birth defects	Outcome	(Outcome confirmation date: Year Month Day)<br>□ Resolved □ Improved □ Not resolved<br>□ Resolved with sequelae □ Death Note) □ Unknown<br>Note) Please consider the outcome as death only if this event was the cause of death. Also, please record the cause of death in 'In case of death' at the bottom of page 10. Please select 'Not resolved' for all unresolved events at the time of death.
		treatment drug・Therapy	□ No □ Yes □ Unknown<br>※ If 'Yes', please fill in the details in 【Concomitant therapy for diseases other than the primary disease during the observation period】 (pages 4-6).	Other than this drug Suspected drug (Supplement)	□ No □ Yes ↓ □ Unknown<br>Select from dropdown
		For this drug Regarding Treatment	□ Discontinuation □ Drug holiday<br>□ Dose reduction □ Dose increase<br>□ No change in administration<br>□ Treatment at another medical institution<br>※ In case of discontinuation, please fill in the details in 【Status of administration of this drug at the end of the observation period】 - Patient outcome (page 2)	Factors other than drugs (Supplement)	□ No □ Yes ↓ □ Unknown
Onset Date : Year Month Day)		Relationship with this drug	□ Yes □ No		

Supplementary description

- If there are other suspected drugs, please record the drug name and fill in the details in 【Concomitant therapy】 (pages 4-6). If 'unrelated' is selected for the relationship with this drug, please be sure to record the reason for the judgment in 'Other suspected drugs' or 'Factors other than drugs'.
- In the 'Treatment for this drug' section, 'discontinuation' refers to cases where treatment with this drug at the medical institution is inevitably abandoned due to progression/worsening of the primary disease, occurrence of adverse events, patient request, death, transfer, etc. 'Drug holiday' refers to cases where administration of this drug was interrupted after the previous administration but was resumed during the observation period. If administration was resumed with a reduced dose after interruption, please select 'Dose reduction'.

【 Safety assessment 】 - Adverse events (continued)

No.	Name of event	In the case of a serious event, please confirm the applicable reason from the 'Severity Criteria' and select the 'Applicable No.'.			
3		Seriousness	□ Non-serious<br>Serious ↓<br>□ No.1: Died<br>□ No.2: Life threatening<br>□ No.3: hitch<br>□ No.4: Hospitalization/prolongation of hospitalization<br>□ No.5: Serious as per No.1-4<br>□ No.6: birth defects	Outcome	(Outcome confirmation date: Year Month Day)<br>□ Resolved □ Improved □ Not resolved<br>□ Resolved with sequelae □ Death Note) □ Unknown
					Note) Only mark the outcome as death if this event was the cause of death. Also, please record the cause of death at the bottom of page 10 under ""In case of death"". Select ""Not recovered"" for all unresolved events at the time of death.
		treatment drug ・Therapy	□ No □ Yes □ Unknown<br>※ If yes, please provide details in 【Concomitant therapy for diseases other than the primary disease during the observation period】 (pages 4-6).	Other than this drug Suspected drug (Supplement)	□ No □ Yes↓ □ Unknown<br>Select from dropdown
		Regarding this drug Treatment Treatment	□ Discontinued □ Drug holiday<br>□ Dose reduction □ Dose increase<br>□ No change in administration<br>□ Treatment at another medical institution<br>※ If discontinued, please provide details in 【Status of administration of this drug at the end of the observation period】 -Patient outcome (page 2)	Factors other than the drug (Supplement)	□ No □ Yes↓ □ Unknown
Onset Date :	Year Month Day	Relation to this drug	□ Yes □ No		
4		Seriousness	□ Non-serious<br>Serious ↓<br>□ No.1: Died<br>□ No.2: Risk of death<br>□ No.3: hitch<br>□ No.4: Hospitalization/Prolonged hospitalization<br>□ No.5: Serious as per No.1-4<br>□ No.6: birth defects	Outcome	(Outcome confirmation date: Year Month Day)<br>□ Recovered □ Improved □ Not recovered<br>□ Resolved with sequelae □ Death Note) □ Unknown
					Note) Only mark the outcome as death if this event was the cause of death. Also, please record the cause of death at the bottom of page 10 under ""In case of death"". Select ""Not recovered"" for all unresolved events at the time of death.
		treatment drug ・Therapy	□ No □ Yes □ Unknown<br>※ If yes, please provide details in 【Concomitant therapy for diseases other than the primary disease during the observation period】 (pages 4-6).	Other than this drug Suspected drug (Supplement)	□ No □ Yes↓ □ Unknown<br>Select from dropdown
		Regarding this drug Treatment Treatment	□ Discontinued □ Drug holiday<br>□ Dose reduction □ Dose increase<br>□ No change in administration<br>□ Treatment at another medical institution<br>※ If discontinued, please provide details in 【Status of administration of this drug at the end of the observation period】 -Patient outcome (page 2)	Factors other than the drug (Supplement)	□ No □ Yes↓ □ Unknown
Onset Date :	Year Month Day	Relation to this drug	□ Yes □ No		
5		Seriousness	□ Non-serious<br>Serious ↓<br>□ No.1: Died<br>□ No.2: Risk of death<br>□ No.3: hitch<br>□ No.4: Hospitalization/Prolonged hospitalization<br>□ No.5: Serious as per No.1-4<br>□ No.6: birth defects	Outcome	(Outcome confirmation date: Year Month Day)<br>□ Recovered □ Improved □ Not recovered<br>□ Resolved with sequelae □ Death Note) □ Unknown
					Note) Only mark the outcome as death if this event was the cause of death. Also, please record the cause of death at the bottom of page 10 under ""In case of death"". Select ""Not recovered"" for all unresolved events at the time of death.
		treatment drug ・Therapy	□ No □ Yes □ Unknown<br>※ If yes, please provide details in 【Concomitant therapy for diseases other than the primary disease during the observation period】 (pages 4-6).	Other than this drug Suspected drug (Supplement)	□ No □ Yes↓ □ Unknown<br>Select from dropdown
		Regarding this drug Treatment Treatment	□ Discontinued □ Drug holiday<br>□ Dose reduction □ Dose increase<br>□ No change in administration<br>□ Treatment at another medical institution<br>※ If discontinued, please provide details in 【Status of administration of this drug at the end of the observation period】 -Patient outcome (page 2)	Factors other than the drug (Supplement)	□ No □ Yes↓ □ Unknown
Onset Date :	Year Month Day	Relation to this drug	□ Yes □ No		

Supplementary description: If other suspected drugs are ""Yes"", please record the drug name and provide details in 【Concomitant therapy】 (pages 4-6). If ""No"" is selected for relation to this drug, please record the reason in ""Other suspected drugs"" or ""Factors other than the drug"".

【 Safety assessment 】 - Adverse events (continued)

No.	Name of event	For serious events, confirm the applicable reason from the ""Seriousness criteria"" and select the ""Applicable No."".			
6		Seriousness	□ Non-serious<br>Serious ↓<br>□ No.1: Died<br>□ No.2: Risk of death<br>□ No.3: hitch<br>□ No.4: Hospitalization/Prolonged hospitalization<br>□ No.5: Serious as per No.1-4<br>□ No.6: birth defects	Outcome	(Outcome confirmation date: Year Month Day)<br>□ Recovered □ Improved □ Not recovered<br>□ Resolved with sequelae □ Death Note) □ Unknown
				Note) Only mark the outcome as death if this event was the cause of death. Also, please record the cause of death at the bottom of page 10 under ""In case of death"". Select ""Not recovered"" for all unresolved events at the time of death.	
		treatment drug・Therapy	□ No □ Yes □ Unknown<br>※ If yes, please provide details in 【Concomitant therapy for diseases other than the primary disease during the observation period】 (pages 4-6).	Other than this drug Suspected drug (Supplement)	□ No □ Yes↓ □ Unknown<br>Select from dropdown
		Regarding this drug Treatment Treatment	□ Discontinued □ Drug holiday<br>□ Dose reduction □ Dose increase<br>□ No change in administration<br>□ Treatment at another medical institution<br>※ If discontinued, please provide details in 【Status of administration of this drug at the end of the observation period】 -Patient outcome (page 2)	Factors other than the drug (Supplement)	□ No □ Yes↓ □ Unknown
Onset Date : Year Month Day		Relation to this drug	□ Yes □ No		
7		Seriousness	□ Non-serious<br>Serious ↓<br>□ No.1: Died<br>□ No.2: Risk of death<br>□ No.3: hitch<br>□ No.4: Hospitalization/Prolonged hospitalization<br>□ No.5: Serious as per No.1-4<br>□ No.6: birth defects	Outcome	(Outcome confirmation date: Year Month Day)<br>□ Recovered □ Improved □ Not recovered<br>□ Resolved with sequelae □ Death Note) □ Unknown
				Note) Only mark the outcome as death if this event was the cause of death. Also, please record the cause of death at the bottom of page 10 under ""In case of death"". Select ""Not recovered"" for all unresolved events at the time of death.	
		treatment drug・Therapy	□ No □ Yes □ Unknown<br>※ If yes, please provide details in 【Concomitant therapy for diseases other than the primary disease during the observation period】 (pages 4-6).	Other than this drug Suspected drug (Supplement)	□ No □ Yes↓ □ Unknown
		Regarding this drug Treatment Treatment	□ Discontinued □ Drug holiday<br>□ Dose reduction □ Dose increase<br>□ No change in administration<br>□ Treatment at another medical institution<br>※ If discontinued, please provide details in 【Status of administration of this drug at the end of the observation period】 -Patient outcome (page 2)	Factors other than the drug (Supplement)	□ No □ Yes↓ □ Unknown
Onset Date : Year Month Day		Relation to this drug	□ Yes □ No		

Supplementary description: If other suspected drugs are ""Yes"", please record the drug name and provide details in 【Concomitant therapy】 (pages 4-6). If ""No"" is selected for relation to this drug, please record the reason in ""Other suspected drugs"" or ""Factors other than the drug"".

In case of death **Please record the cause of death and autopsy. Ensure the cause of death matches the event name recorded for the applicable adverse event.**

Date of death	Year Month Day	Investigations post mortem	to perform ⇒<br>□ Not conducted<br>□ Unknown	autopsy finding :
cause of death	Select from dropdown			

For adverse events judged as ""No"" causal relationship with this drug above, please record the reason for denial in the comments field.

comments field	

If ""Treatment at another medical institution"" was selected as the action for the adverse event above, please record the following information.

Which medical institution is providing treatment?	□ Hospital name ( ) □ Unknown
Is it permissible to confirm details with the physician at that medical institution?	□ Permitted □ Not Permitted

【 test 】 - Related to adverse events

- Please record the results of clinical tests related to adverse events. At that time, also record the clinical test results from before administration of this drug, from onset to outcome confirmation (including the worst test values).

No.	Timepoint / Test items and units		Baseline	After administration Course from onset of the event to outcome confirmation (including the worst test values)				
			years month day	years month day	years month day	years month day	years month day	years month day
1								
2								
3								
4								
5								
6								
7								
8								
9								
10								
11								
12								
13								
14								
15								
16								
17								
18								
19								
20								

【 hyponatraemia 】 - Investigation items at the time of hyponatremia onset

- If the adverse event ""Hyponatremia"" occurs, please record the following conditions at the time of onset.
- This information is necessary for the assessment of hyponatremia and will be used for cause analysis, so please obtain and record the following information from the patient: presence of symptoms (fatigue, headache, nausea/vomiting, confusion, edema, seizures, stupor/coma, etc.), presence of acute diseases at onset (systemic infections, fever, gastroenteritis, etc.).

Subgroup	which way the wind blows
Fluid intake status before and after administration of this drug (oral intake, drip/infusion)	(Please record specific situations)
Daily urine volume before and after hyponatremia onset	20 Year Month Day mL □ Not measured<br>20 Year Month Day mL □ Not measured
presence of symptoms	□ No<br>Yes ⇒ (Please record specific symptoms)
Presence of acute disease at onset	□ No<br>Yes ⇒ (Please record specific symptoms)
Dinner time and administration time of this drug immediately before onset (24Hr)	Dinner time: Hour Minute<br>Administration time of this drug: Hour Minute
